# Association between longitudinal dietary patterns and changes in obesity: a population-based cohort study

**DOI:** 10.3389/fpubh.2023.1227994

**Published:** 2023-12-01

**Authors:** Liuyan Zheng, Xinyue Lu, Jianhui Guo, Xingyan Xu, Le Yang, Xiaoxu Xie, Huangyuan Li, Siying Wu

**Affiliations:** ^1^Department of Epidemiology and Health Statistics, School of Public Health, Fujian Medical University, Fuzhou, Fujian, China; ^2^Institute of Child and Adolescent Health, School of Public Health, Peking University, Beijing, China; ^3^Department of Preventive Medicine, School of Public Health, Fujian Medical University, Fuzhou, Fujian, China

**Keywords:** low-carbohydrate diet, low-fat diet, multitrajectories, obesity change, body mass index, China

## Abstract

**Introduction:**

Research on the trajectory of dietary patterns and changes in obesity has been inconclusive.

**Methods:**

This study described the dietary intake and adiposity trajectories of Chinese adults and assessed the association between dietary trajectories and changes in body mass index (BMI) and waist-to-hip ratio (WHR). We used data from 3, 643 adults who participated in the China Health and Nutrition Survey from 1997 to 2015. Detailed dietary data were collected by conducting three consecutive 24-h recalls. Multitrajectories of diet scores were identified by a group-based multitrajectory method. We described the change in BMI and WHR using group-based trajectory modeling. We assessed the associations between dietary trajectories and changes in people with obesity using a logistic regression model.

**Results:**

Our study revealed four trajectories of low-carbohydrate (LCD) and low-fat diet (LFD) scores. Three adiposity trajectories were identified according to the baseline level and developmental trend of BMI and WHR. Compared with the reference group, which was characterized by sustained healthy dietary habits with healthy diet scores at baseline and sustained maintenance of healthy diet scores, the other three diet trajectories had a higher risk of falling into the adverse adiposity trajectory.

**Discussion:**

Maintaining a healthy LCD and LFD can markedly decrease the risk of adiposity.

## 1 Introduction

Obesity is a growing public health problem in which excess body fat has accumulated, and it poses a potential risk to an individual's health (1). Abdominal obesity, a distinctive form of obesity, constitutes a key element of metabolic syndrome (2). Numerous non-communicable diseases have been linked to obesity and abdominal obesity, such as cancer (3, 4), cardiovascular disease (5, 6), type 2 diabetes (6), and chronic kidney disease (7). Epidemiological data published by the World Health Organization show that the global prevalence of adiposity almost tripled between 1975 and 2016, and more than 650 million adults were estimated to be classified as obese in 2016 (1). The global spread of adiposity has been labeled a pandemic (8). This phenomenon was once seen as a problem only in upper-income countries but has been on the rise in low- and middle-income countries for many years (9). By 2018, ~85 million Chinese individuals aged 18–69 were obese, three times the number in 2004 (10). The latest national prevalence figures for 2015–2019, using Chinese criteria, show that 34.3% of adults (aged 18 and over) in China are classified as overweight, while 16.4% are classified as obese (11). Moreover, 4.7 million people died prematurely with adiposity based on the Global Burden of Disease 2017 (12).

Obesity is the result of a mismatch between energy intake and energy expenditure. Diet, as the primary source of energy intake, plays a crucial role in the onset and progression of obesity. A large body of evidence suggests that it is the quality, not the quantity, of carbohydrates and fats that define disease and health outcomes (13–15). The Mediterranean diet is presently recommended as a management strategy for weight loss (16), and existing research shows that adherence to the Dietary Approaches to Stop Hypertension (DASH) diet is associated with a reduced risk of overweight and obesity in Iranian women (17). The common feature of both of these dietary patterns is that they are both varieties of “plant-based diets” that contain natural foods and less ultra-processed foods (18). But whether other prevailing dietary patterns that focus on fat and carbohydrates, including low-carbohydrate diets (LCDs) or low-fat diets (LFDs) can promote weight loss remains controversial. Individuals who follow LCDs restrict carbohydrates to increase their intake of fats and/or proteins, while those who follow LFDs restrict fats to increase carbohydrates. There are controversial viewpoints between LCDs and LFDs regarding weight loss (19–24).

This is probably due to inconsistencies in the definitions of LCD and LFD. Some studies (25, 26) have defined LCD or LFD, depending on the proportion of carbohydrate or fat intake to daily calorie intake, low-carbohydrate and low-fat diets can further reduce body weight by reducing liver volume. Other researchers have examined the different sources of carbohydrates, fats and proteins in diets over the past decades (20, 27). One of these studies (20), conducted in Iran, found no significant association between low-carbohydrate and low-fat diets and overweight or obesity. In China, dietary macronutrient intake has not remained static, but rather has changed over time. For example, fat consumption spiked from 1982 to 2012, while the estimated proportion of energy intake from carbohydrates declined (28). Moreover, little research has been conducted to explore the association between dietary trajectories and changes in adiposity based on follow-up over time. It remains unclear how the dynamics of dietary trajectories play a role in adiposity progression.

Unlike previous studies that analyzed dietary intake status based on single-point assessment, we describe distinct trajectories of LCDs and LFDs using data published from 1997 to 2015 in the China Health and Nutrition Survey (CHNS). We also analyzed the transition between normal weight and overweight during 15 years of follow-up in the current study. In addition, we examined the associations between these dietary trajectories and changes in BMI and WHR. We intended to investigate (1) the trajectories of dietary intake and adiposity in Chinese adults from 1997 to 2015; (2) the associations between dietary trajectories and the variations in BMI and WHR.

## 2 Materials and methods

### 2.1 Study participants

In this study, we utilized data collected by the CHNS from 1997 to 2015. The CHNS aims to gather representative information on important risk factors for public health, health outcomes, and the state of nutrition in Chinese communities (28). Since its establishment in 1989, the CHNS has been followed up every 2–4 years. A total of ten waves of data have been published, covering the years 1989, 1991, 1993, 1997, 2000, 2004, 2006, 2009, 2011, and 2015. The surveys were authorized by the Institutional Review Boards of the University of North Carolina at Chapel Hill and the National Institute for Nutrition and Health, Chinese Centre for Disease Control and Prevention, and all participants gave valid informed consent. Details of the design and procedures of the CHNS have been reported (29).

Our analysis utilized data from nine waves of the CHNS spanning from 1997 to 2015. The 1989 wave was excluded as it did not include dietary assessment data for all participants. The 1991 and 1993 waves were excluded from the analysis due to inconsistencies in dietary coding compared to other years. It should be noted that the dietary data from the 2015 wave has not yet been fully published, so only the available data, such as the 2015 physical examination data, were used in our study. Participants with less than two waves of dietary data, body mass index (BMI) value and waist–hip ratio data, with extreme total dietary energy intakes (<800 or >6,000 kcal/d for males; <600 or >4,000 kcal/d for females) (30), with general and abdominal adiposity at baseline, and with missing covariates in all follow-up surveys in which they participated were excluded from the analysis. Participants who were pregnant, lactating, or younger than 18-year-old were also excluded. The final analysis included 3,643 study participants. Supplementary Figure S1 illustrates the process of selecting study participants.

### 2.2 Evaluation of LCD and LFD scores

The dietary scores used in this study were calculated from dietary information collected from the CHNS dataset. A 24-h dietary recall was used for each wave of the CHNS to gather dietary intake data from Chinese adults over three consecutive days, namely, two workdays and one weekend day. Household food consumption data were also collected over the same 3-day period. How detailed dietary data are collected and allocated in the CHNS has been described elsewhere (9, 31).

To reduce bias arising from underreporting of food consumption and to represent dietary ingredients, we calculated the LCD and LFD scores based on the percentage of energy intake rather than absolute intake (13, 32, 33). First, we categorized participants by gender. Second, we further divided the participants into 11 groups based on their energy percentage derived from fat, protein, and carbohydrates. To evaluate LCD, a scoring system was developed wherein individuals with the highest fat and protein intake in each stratum were awarded 10 points, while those with the lowest intake were given 0 points. The order of the layers was reversed for carbohydrates. The scores for the three macronutrients were then aggregated to produce the overall LCD score, which ranged from 0 to 30. To establish unhealthy and healthy LFD scores, similar approaches were used. Unhealthy LCD was determined based on the percentage of energy accounted for by high-quality carbohydrates, saturated fats, and animal protein, while the healthy LCD was calculated using low-quality carbohydrates, unsaturated fats, and plant protein as determining factors (Supplementary Table S1). Similar methods were used to compute both unhealthy and healthy LFD scores in our study (Supplementary Table S2). The distribution of energy percentage criteria for determining scores for low-carbohydrate and low-fat diets in men was presented in Supplementary Table S3. The distribution of energy percentage criteria for determining scores for low-carbohydrate and low-fat diets in women was presented in Supplementary Table S4.

### 2.3 Anthropometric variables

Adiposity was our outcome variable of interest. Standardized procedures were used by well-trained health workers to measure participants' height (Model 206, SECA), weight (Model 880, SECA), and waist circumference (WC) in CHNS. Weight (in kilograms) divided by height (in meter) squared equals BMI. Based on the cut-off values recommended by the Working Group on Obesity in China, BMI was classified into four categories in our study (underweight: BMI < 18.5; normal: 18.5 ≤ BMI < 24.0; overweight: 24.0 ≤ BMI < 28.0; and obesity: BMI ≥ 28.0) (34) and assigned ascending values (1, 2, 3, 4). The second major outcome variable was abdominal obesity, which was defined as a waist-to-hip ratio (WHR) of ≥0.9 in males or ≥0.85 in females (35). For the non-abdominal obesity group, a score of 1 was assigned, and abdominal obesity was scored as 2. WHR was determined by dividing waist circumference by hip circumference. WC was measured at the end of exhalation at a midpoint between the top of the iliac crest and the bottom of the rib cage in CHNS. HC was taken at the level of maximum gluteal protrusion. The WC and HC were both measured with a SECA tape, accurate to the nearest 0.1 cm (36). Furthermore, we described the change in BMI and WHR using group-based trajectory modeling.

### 2.4 Covariates

In our study analysis, we incorporated two categories of diet-related and adiposity-related confounders, sociodemographic factors including sex, age, marital status, nationality, education, family economic level, geographic location, lifestyle factors including smoking, drinking, and physical activity (PA), and dietary energy. Marital status was classified into three groups: married, unmarried, and divorced/separated/widowed. Nationality was divided into Han and non-Han. Education level was categorized into three types: junior high school or below, senior high school, and college or above. Participants' yearly household income per capita at baseline was divided into three categories (low: <1,369.68$, middle: 1,369.68–2,739.35$, and high: >2,739.35$) based on the per capita annual family income tertiles. The geographic location was divided into four clusters: Liaoning, Heilongjiang, Jiangsu, and Shandong provinces (37). Provincial boundaries in China are based on a combination of cultural practices, topographical features and latitude and longitude. As a result, there are differences in food consumption patterns between geographical locations. Participants were classified as current smokers, ex-smokers and never drinkers, and current drinkers and never drinkers based on their recent smoking and drinking status, respectively. Participants' physical activity level was classified into three types (light, medium, and heavy) according to their self-reported activities, including occupational, domestic, transportation, and recreational sports activities. Total physical activity intensity is determined by a calculation based on metabolic equivalents, which takes into account the cumulative time spent each week in different physical activities, including those related to work, household chores, transport and leisure. The weekly scores for different types of physical activity are derived by multiplying the weekly frequency by the time spent per day. The resulting product, obtained by multiplying metabolic equivalents and duration and summing, represents the total amount of physical activity (38). In accordance with guidelines from the World Health Organization (39), participants were categorized into three groups based on their total weekly physical activity levels: (1) Light physical activity (<600 METs-min/week), (2) Moderate physical activity (600–1,199 METs-min/week), and (3) Heavy physical activity (≥1,200 METs-min/week).

### 2.5 Statistical analysis

Continuous variables were expressed as medians (25th percentile, 75th percentile) due to non-normal distribution. Classification variables are presented as the number of people (%). The Mann-Whitney *U*-test and chi-square tests were used for comparison of continuous and categorical variables, respectively.

To analyze the trajectories of the LCD and LFD scores, including HLCD, ULCD, HLFD, and ULFD, and to classify them into four categories, we used group-based multitrajectory modeling (40). To illustrate the likelihood of subjects maintaining HLCD, ULCD, HLFD, and ULFD simultaneously over time, we created an annual model using a STATA multitrajectory modeling plugin (41), with trajectories defined based on the year as the time scale. Rigorous criteria were applied to determine the best-fitting model in the statistical analysis. (1) We utilized the Bayesian information criterion (BIC), with the lowest value indicating the best fit, and evaluated the percentage change in BIC to choose between more complex (adding a specific set of trajectories) and simpler models. (2) We enrolled at least 5% of the study participants in each trajectory category to guarantee an adequate sample size. (3) We determined the average posterior probability of membership within each group, with values >0.7 indicating satisfactory internal reliability (42). A group-based trajectory model was used to classify adiposity and abdominal obesity. The basic requirements for modeling using this approach are the same as those outlined above. After identifying the trajectories of the scores, we generated categorical variables to designate the trajectory categories of each object and subsequently incorporated this variable into our multinomial logistic regression models. Then, multinomial logistic regression models were used to separately analyze the association between the BMI or WHR trajectory and the change trajectory patterns of LCD and LFD scores, with effects reported as odds ratios (*OR*) and 95% confidence intervals (*CI*).

Last, considering that the exclusion of participants who had diabetes at baseline or who had diabetes at baseline and all follow-up, years may affect our results, we conducted a sensitivity analysis in this population to consolidate our findings. To account for the potential selection bias of subject screening, we also performed inverse probability weighting (IPW). Further assessment of the impact of missing covariate data on the association between the change in LCDs or LFDs and adiposity trajectories and sensitivity analyses of the missing value samples was performed using the multiple imputation method. All analyses were performed in Stata (version 17.0) and R (version 4.2.2) software, and a two-sided test of *P* < 0.05 indicated statistical significance.

## 3 Results

Following the initial screening process, our study included 3,643 participants, all of whom were enrolled two or more times between 1997 and 2015. Table 1 lists the characteristics of the study attendees according to adiposity trajectory group. Participants with an upwards overweight trajectory more often had a diet trajectory of deteriorating dietary habits, and a lower education level, and were non-smokers. Table 2 presents the characteristics of the study participants according to the abdominal obesity trajectory group.

**Table 1:** Characteristics of study participants by adiposity trajectory group.

**Characteristics**	**Overall**	**Normal weight**	**Overweight trajectory**		**Overweight upwards trajectory**	
	**(*n* = 3, 643)**	**(*n* = 2, 115)**	**(*n* = 1, 243)**	***P* ^a^**	**(*n* = 285)**	***P* ^b^**
**Trajectories**				0.001		0.008
Group2, sustained healthy dietary habits	909 (25.0)	554 (27.5)	288 (21.8)		67 (21.9)	
Group1, improved dietary habits	931 (25.6)	476 (23.6)	375 (28.4)		80 (26.1)	
Group3, worsening dietary habits	770 (21.1)	433 (21.5)	285 (21.6)		52 (17.0)	
Group4, deteriorating dietary habits	1,033 (28.4)	552 (27.4)	374 (28.3)		107 (35.0)	
**Age (years)**				< 0.001		0.446
≤ 45	2,321 (63.7)	1,314 (65.2)	818 (61.9)		189 (61.8)	
45–60	939 (25.8)	470 (23.3)	388 (29.3)		81 (26.5)	
≥60	383 (10.5)	231 (11.5)	116 (8.8)		36 (11.8)	
**Sex**				0.236		0.064
Male	1,896 (52.0)	1,043 (51.8)	712 (53.9)		141 (46.1)	
Female	1,747 (48.0)	972 (48.2)	610 (46.1)		165 (53.9)	
**Nationalities**				0.302		0.111
Han	3,306 (90.7)	1,841 (91.4)	1,194 (90.3)		271 (88.6)	
Non-han	337 (9.3)	174 (8.6)	128 (9.7)		35 (11.4)	
**Annual per capita family income**				0.894		0.722
Low (< 1,369.68$)	1,138 (31.2)	624 (31.0)	418 (31.6)		96 (31.4)	
Middle (1,369.68–2,739.35 $)	1,100 (30.2)	617 (30.6)	396 (30.0)		87 (28.4)	
High (>2,739.35$)	1,405 (38.6)	774 (38.4)	508 (38.4)		123 (40.2)	
**Geographic location**				< 0.001		< 0.001
Liaoning	1,205 (33.1)	581 (28.8)	473 (35.8)		151 (49.3)	
Heilongjiang	938 (25.7)	571 (28.3)	323 (24.4)		44 (14.4)	
Jiangsu	984 (27.0)	622 (30.9)	308 (23.3)		54 (17.6)	
Shandong	516 (14.2)	241 (12.0)	218 (16.5)		57 (18.6)	
**Marital status**				0.005		0.013
Married	3,128 (85.9)	1,709 (84.8)	1,149 (86.9)		270 (88.2)	
Unmarried	108 (3.0)	80 (4.0)	26 (2.0)		2 (0.7)	
Divorced/separate/widowed	407 (11.2)	226 (11.2)	147 (11.1)		34 (11.1)	
**Years of education (years)**				< 0.001		0.065
< 9	2,688 (73.8)	1,486 (73.7)	961 (72.7)		241 (78.8)	
9–12	637 (17.5)	323 (16.0)	268 (20.3)		46 (15.0)	
>12	318 (8.7)	206 (10.2)	93 (7.0)		19 (6.2)	
**Drinking status**				0.090		0.043
Current drinker	1,973 (54.2)	1,080 (53.6)	748 (56.6)		145 (47.4)	
Never	1,670 (45.8)	935 (46.4)	574 (43.4)		161 (52.6)	
**Physical activities**				0.006		0.009
Light	152 (4.2)	89 (4.4)	49 (3.7)		14 (4.6)	
Medium	466 (12.8)	221 (11.0)	193 (14.6)		52 (17.0)	
Heavy	3,025 (83.0)	1,705 (84.6)	1,080 (81.7)		240 (78.4)	
**Smoking status**				0.009		0.009
Current smoker	1,060 (29.1)	632 (31.4)	358 (27.1)		70 (22.9)	
Ex-smoker	558 (15.3)	287 (14.2)	226 (17.1)		45 (14.7)	
Never	2,025 (55.6)	1,096 (54.4)	738 (55.8)		191 (62.4)	
**Energy (kcal/d)**	1,762.5 (1,479.9, 2,074.0)	1,748.8 (1,467.3, 2,078.4)	1,783.3 (1,502.2, 2,073.0)	0.076	1,769.5 (1,487.9, 2,076.5)	0.658

a The difference test between the “Overweight” trajectory group and the “Normal weight” trajectory group.

b The difference test between the “Overweight upwards trajectory” trajectory group and the “Normal weight” trajectory group.

Energy is presented as number (proportion %). The others are presented as median (IQR). *P*-values were calculated using analysis of the Mann-Whitney *U*-test and Chi-square test for continuous and categorical variables, respectively.

**Table 2:** Characteristics of study participants by abdominal obesity trajectory group.

**Characteristics**	**Overall**	**No abdominal obesity**	**Slowly growth of abdominal obesity**		**Rapidly growth of abdominal obesity**	
	**(*n* = 3, 643)**	**(*n* = 2, 104)**	**(*n* = 938)**	***P* ^a^**	**(*n* = 601)**	***P* ^b^**
**Trajectories**				0.019		0.022
Group2, sustained healthy dietary habits	909 (25.0)	417 (25.7)	181 (26.8)		311 (23.1)	
Group1, improved dietary habits	931 (25.6)	403 (24.8)	163 (24.1)		365 (27.1)	
Group3, worsening dietary habits	770 (21.1)	377 (23.2)	122 (18.1)		271 (20.1)	
Group4, deteriorating dietary habits	1,033 (28.4)	426 (26.2)	209 (31.0)		398 (29.6)	
**Age (years)**				0.005		< 0.001
≤ 45	2,321 (63.7)	1,129 (69.6)	472 (69.9)		720 (53.5)	
45–60	939 (25.8)	342 (21.1)	165 (24.4)		432 (32.1)	
≥60	383 (10.5)	152 (9.4)	38 (5.6)		193 (14.3)	
**Sex**				0.317		< 0.001
Male	1,896 (52.0)	917 (56.5)	366 (54.2)		613 (45.6)	
Female	1,747 (48.0)	706 (43.5)	309 (45.8)		732 (54.4)	
**Nationalities**				0.316		0.038
Han	3,306 (90.7)	1,490 (91.8)	611 (90.5)		1,205 (89.6)	
Non-han	337 (9.3)	133 (8.2)	64 (9.5)		140 (10.4)	
**Annual per capita family income**				0.112		0.365
Low (< 1,369.68$)	1,138 (31.2)	502 (30.9)	188 (27.9)		448 (33.3)	
Middle (1,369.68–2,739.35$)	1,100 (30.2)	498 (30.7)	197 (29.2)		405 (30.1)	
High (>2,739.35$)	1,405 (38.6)	623 (38.4)	290 (43.0)		492 (36.6)	
**Geographic location**				0.977		< 0.001
Liaoning	1,205 (33.1)	458 (28.2)	193 (28.6)		554 (41.2)	
Heilongjiang	938 (25.7)	451 (27.8)	185 (27.4)		302 (22.5)	
Jiangsu	984 (27.0)	462 (28.5)	196 (29.0)		326 (24.2)	
Shandong	516 (14.2)	252 (15.5)	101 (15.0)		163 (12.1)	
**Marital status**				< 0.001		< 0.001
Married	3,128 (85.9)	1,387 (85.5)	613 (90.8)		1,128 (83.9)	
Unmarried	108 (3.0)	73 (4.5)	6 (0.9)		29 (2.2)	
Divorced/separate/widowed	407 (11.2)	163 (10.0)	56 (8.3)		188 (14.0)	
**Years of education (years)**				0.851		0.187
< 9	2,688 (73.8)	1,182 (72.8)	489 (72.4)		1,017 (75.6)	
9–12	637 (17.5)	289 (17.8)	126 (18.7)		222 (16.5)	
>12	318 (8.7)	152 (9.4)	60 (8.9)		106 (7.9)	
**Drinking status**				0.474		< 0.001
Current drinker	1,973 (54.2)	940 (57.9)	380 (56.3)		653 (48.6)	
Never	1,670 (45.8)	683 (42.1)	295 (43.7)		692 (51.4)	
**Physical activities**				0.007		< 0.001
Light	152 (4.2)	60 (3.7)	21 (3.1)		71 (5.3)	
Medium	466 (12.8)	189 (11.6)	50 (7.4)		227 (16.9)	
Heavy	3,025 (83.0)	1,374 (84.7)	604 (89.5)		1,047 (77.8)	
**Smoking status**				0.046		< 0.001
Current smoker	1,060 (29.1)	535 (33.0)	191 (28.3)		334 (24.8)	
Ex-smoker	558 (15.3)	224 (13.8)	112 (16.6)		222 (16.5)	
Never	2,025 (55.6)	864 (53.2)	372 (55.1)		789 (58.7)	
**Energy (kcal/d)**	1,762.5 (1,479.9, 2,074.0)	1,789.9 (1,497.5, 2,096.1)	1,818.5 (1,540.1, 2,128.5)	0.007	1,714.6 (1,438.1, 2,007.3)	< 0.001

a The difference test between the “Slowly growth of abdominal obesity” trajectory group and the “No abdominal obesity” trajectory group.

b The difference test between the “Rapidly growth of abdominal obesity” trajectory group and the “No abdominal obesity” trajectory group.

Data are presented as median (IQR) or number (proportion %). *P*-values were calculated using analysis of the Mann-Whitney *U-*test and Chi-square test for continuous and categorical variables, respectively.

Compared with the no-abdominal-obesity group, the rapid growth of abdominal obesity had a higher proportion of deteriorating dietary habits, in older adults. They were more likely to be non-drinkers and non-smokers. The flowcharts depicting the process of sample selection can be found in Supplementary Figure S1.

### 3.1 Assessments of multi-trajectories of LCD and LFD scores

**Figure 1:**
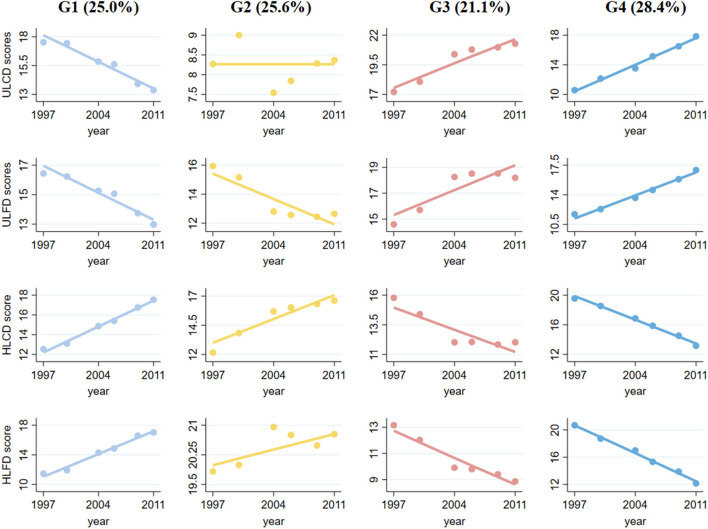
Multitrajectories of low-carbohydrate and low-fat diet scores among study populations. Source: CHNS 1997–2011. Solid lines represent the average estimated low-carbohydrate and low-fat diet scores over time. The dots represent the actual data, where we weighted each individual's responses based on posterior probabilities of group membership. Group 1 refers to the participants with unhealthy diet scores who sustained changes for the better; Group 2 refers to the participants with healthy diet scores who sustained changed for the better; Group 3 refers to the participants with moderate diet scores and persistent worsening; and Group 4 refers to the participants with healthy diet scores and persistent worsening.

In the total sample of six waves of CHNS data, we identified four diverse diet score multitrajectories among participants based on the unhealthy LCD score, unhealthy LFD score, healthy LCD score, and healthy LFD score (Figure 1). The overall LCD and LFD score ranged from 0 to 30. A quarter of the participants (25.6%) fell into a diet trajectory characterized by sustained healthy dietary habits (Group 2, reference category). They were identified as having a high healthy LCD score and healthy LFD score, a low unhealthy LCD score at baseline and sustained change for the better, they included persons whose healthy LCD score increased from 12 to 17, whose healthy LFD score slowly increased from 20 to 21, whose unhealthy LCD score fluctuated around 8, and whose unhealthy LFDs score decreased from 16 to 12. A total of 909 participants (25.0%) were able to maintain improved dietary habits (Group 1). Of the participants, 770 individuals (21.1%) exhibited worsening dietary habits characterized by suboptimal dietary habits at baseline that persisted over time (Group 3). A total of 1,033 participants (28.4%) had deteriorating dietary habits, characterized by a change from previously good to poor dietary habits, which continued to worsen over time (Group 4).

### 3.2 Assessments of trajectory patterns of adiposity and abdominal obesity

**Figure 2:**
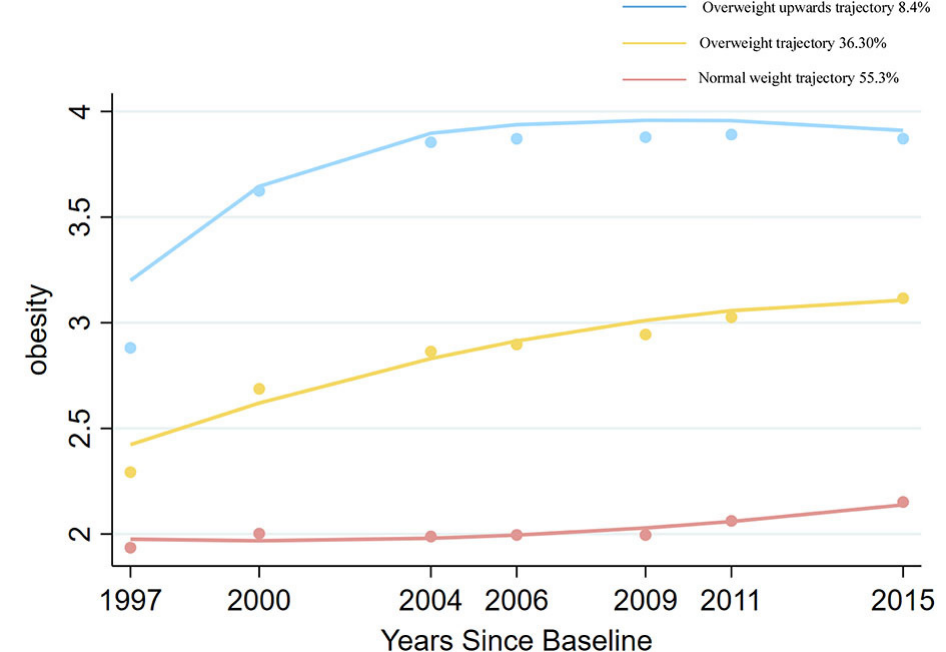
Change trajectories of adiposity measured by BMI groups. Source: CHNS 1997–2015.

**Figure 3:**
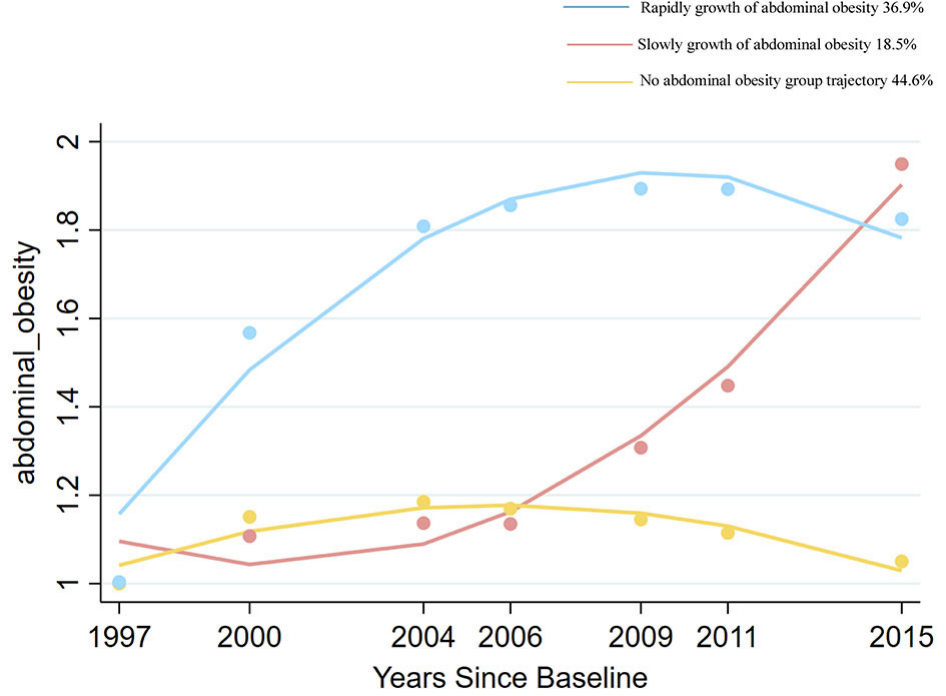
Change trajectories of abdominal obesity measured by waist to hip ratio groups. Source: CHNS 1997–2015.

Figures 2, 3 show adiposity and abdominal obesity from 1997 to 2015. Three patterns of adiposity change trajectory were identified based on the baseline level and rates of change in the BMI groups (Figure 2). The first pattern of adiposity trajectory featuring low stability of normal weight (score 2) during follow-up visits was named the “normal weight trajectory”. The second pattern, characterized by an increase from normal weight to overweight (score from 2.5 to 3) in BMI groups during follow-up visits, was named the “overweight trajectory”. The third pattern, which was characterized by staying stable at around obesity (score 4) in BMI groups during follow-up visits, was named the “overweight upwards trajectory”. We identified three abdominal obesity groups (Figure 3), labeled the “no abdominal-obesity group,” “slow growth of abdominal obesity,” and “rapid growth of abdominal obesity,” respectively, according to the initial level and development trend of trajectories.

### 3.3 Associations between the adiposity trajectory and the change trajectory patterns of LCD and LFD scores

**Figure 4:**
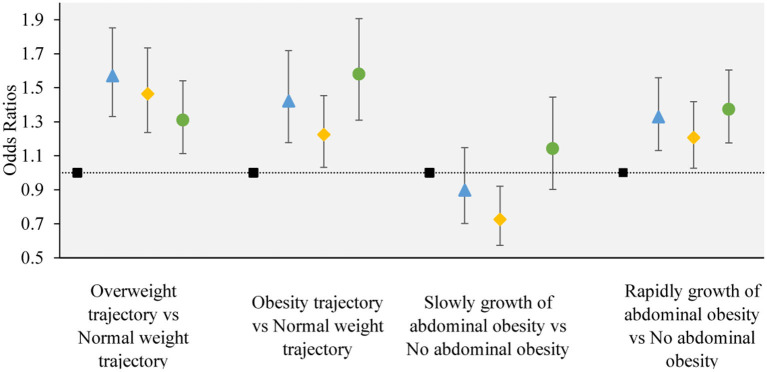
The associations between the obesity or abdominal obesity trajectory and the change trajectory patterns of low-carbohydrate and low-fat diet scores. Source: CHNS 1997–2015. Adjusted model: adjusted for sociodemographic factors, including sex, age, marital status, nationality, education level, family economic level, region, and lifestyle factors, including smoking status, drinking status, physical activity, and dietary energy. ■ refers to the sustained healthy dietary habits; 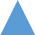 refers to the improved dietary habits; 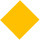 refers to the worsening dietary habits; 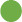 refers to the deteriorating dietary habits.

The results from multinomial logistic regression showed a significant association between the change trajectory patterns of LCD and LFD scores and adverse adiposity trajectory (adverse adiposity trajectory refers to “overweight trajectory” and “overweight upwards trajectory”, “normal weight trajectory” as reference) (Figure 4). The group of participants with sustained healthy dietary habits as the reference category (Group 2), Group 1 (*OR* = 1.570, 95% CI, 1.331–1.852 in overweight; *OR* = 1.422, 95% CI, 1.177–1.718 in people with obesity), Group 3 (*OR* = 1.465, 95% CI, 1.237–1.734 in overweight; *OR* = 1.225, 95% CI, 1.032–1.454 in people with obesity) and Group 4 (*OR* = 1.310, 95% CI, 1.113–1.541 in overweight; *OR* = 1.580, 95% CI, 1.309–1.907 in obesity) significantly increased the risk of “overweight” and “overweight upwards”.

### 3.4 Associations between the abdominal obesity trajectory and the change trajectory patterns of LCD and LFD scores

The change trajectory patterns of LCD and LFD scores were associated with an adverse abdominal obesity trajectory (adverse abdominal obesity trajectory refers to “rapid growth of abdominal obesity”, and “no abdominal obesity” as the reference) (Figure 4). In the multinomial logistic regression analysis, compared with sustained healthy dietary habits, Group 1 (*OR* = 1.328, 95% *CI*, 1.131–1.559), Group 3 (*OR* = 1.207, 95% *CI*, 1.027–1.418), and Group 4 (*OR* = 1.373, 95% *CI*, 1.175–1.604) were associated with a higher degree of risk in “Rapidly growth of abdominal obesity”. compared with sustained healthy dietary habits, Group 3 (*OR* = 0.726, 95% *CI*, 0.573–0.921) was associated with a lower degree of risk of “slow growth of abdominal obesity”. No significant association was observed between other dietary trajectories and the slow growth of abdominal obesity.

### 3.5 Subgroup analysis

**Figure 5:**
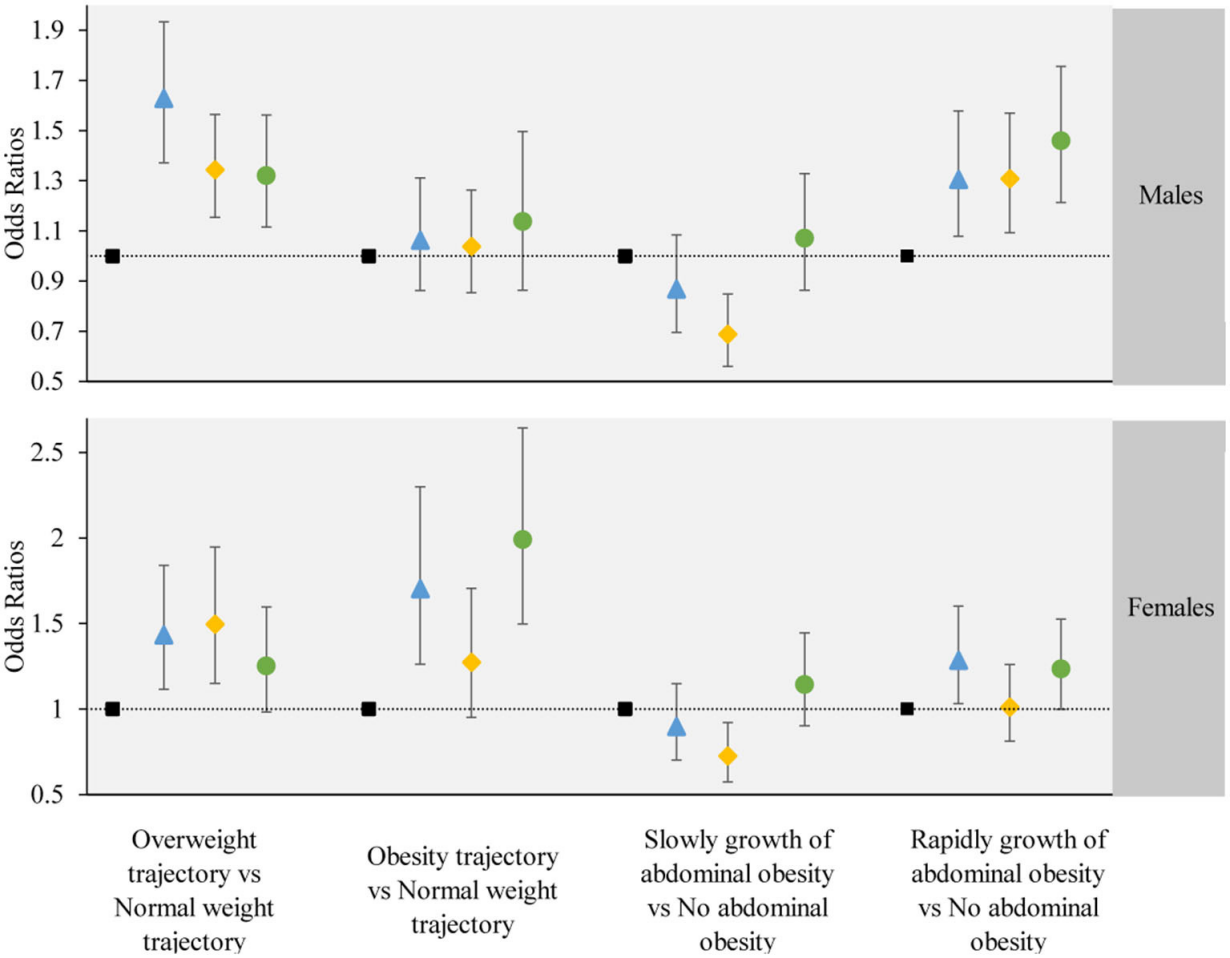
The associations between the obesity or abdominal obesity trajectory and the change trajectory patterns of low-carbohydrate and low-fat diet scores stratified by participant's gender. Source: CHNS 1997–2015. Adjusted model: adjusted for sociodemographic factors, including sex, age, marital status, nationality, education level, family economic level, region, and lifestyle factors, including smoking status, drinking status, physical activity, and dietary energy. ■ refers to the sustained healthy dietary habits; 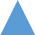 refers to the improved dietary habits; 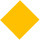 refers to the worsening dietary habits; 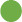 refers to the deteriorating dietary habits.

**Figure 6:**
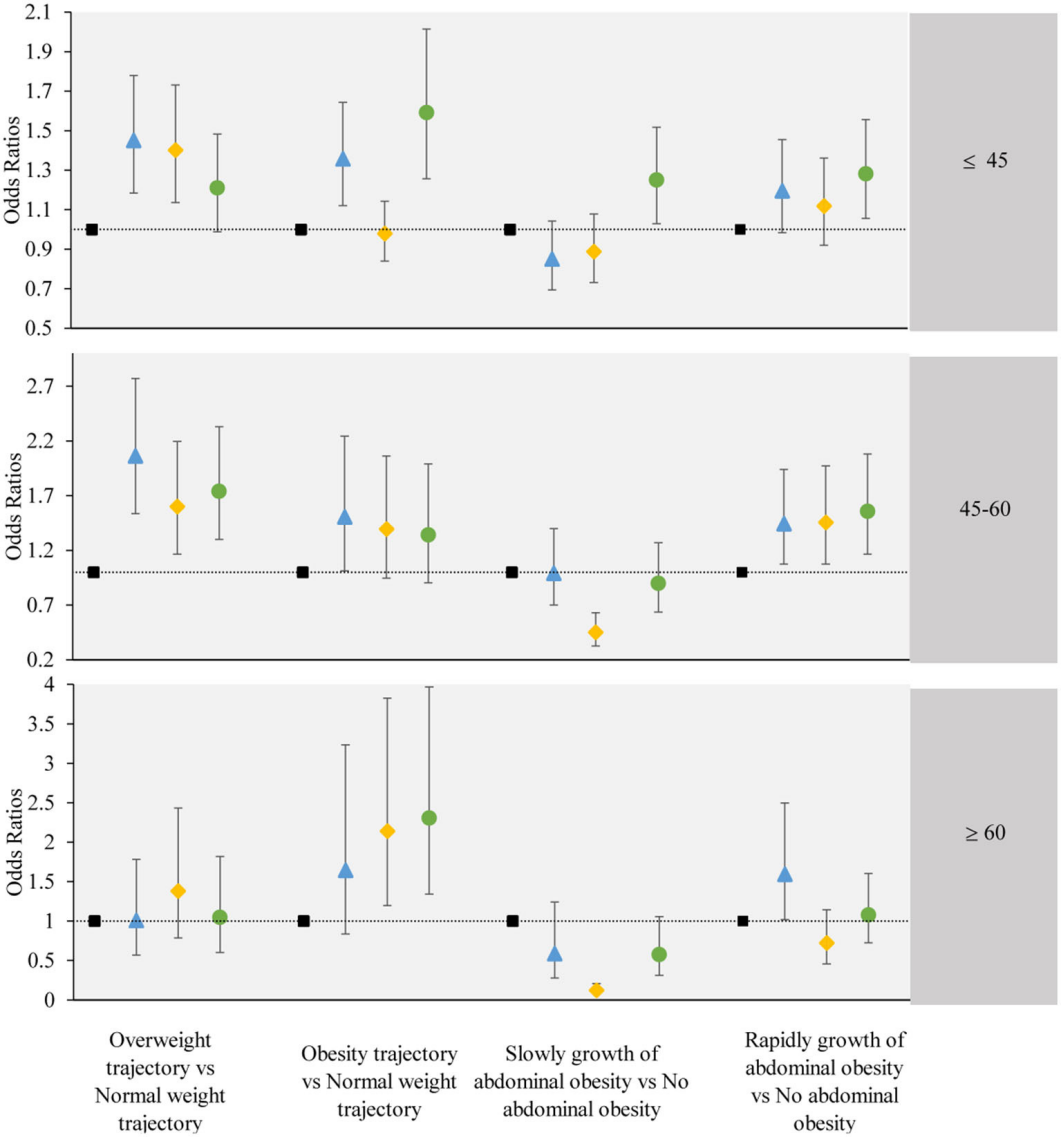
The associations between the obesity or abdominal obesity trajectory and the change trajectory patterns of low-carbohydrate and low-fat diet scores stratified by participant's age. Source: CHNS 1997–2015. Adjusted model: adjusted for sociodemographic factors, including sex, age, marital status, nationality, education level, family economic level, region, and lifestyle factors, including smoking status, drinking status, physical activity, and dietary energy. ■ refers to the sustained healthy dietary habits; 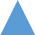 refers to the improved dietary habits; 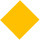 refers to the worsening dietary habits; 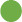 refers to the deteriorating dietary habits.

We further stratified by age and gender, and performed subgroup analysis. The subgroup analysis results were largely consistent with the main findings, except for individual groups which did not demonstrate statistical significance. The results of the subgroup analysis are shown in Figures 5, 6.

### 3.6 Sensitivity analyses

The results of our sensitivity analyses demonstrated the robustness of the main findings. First, in analyses using inverse-probability weighting to minimize the differences in participant characteristics, the analysis results were generally in line with the results of the main analyses (Supplementary Tables S5, S6). Second, following the multiple imputations of missing data, the results remained consistent with those obtained from the analytic samples, with no substantial differences in the direction or magnitude of the observed associations (Supplementary Tables S7, S8). Excluding participants with diabetes at baseline and those with diabetes at both baseline and follow-up years, the results were generally consistent with the main analysis (Supplementary Tables S9–S12).

## 4 Discussion

Using a nationally representative sample of Chinese adults, this study identified three obesity and abdominal obesity trajectories that can dynamically assess the changes in obesity status in the Chinese population. In addition, four various trajectories of LCD and LFD scores were drawn according to the total and type of macronutrients. Approximately a quarter of the participants had healthy diet scores at baseline and sustained healthy diet scores, and the risk of adiposity was lowest in this group. The other diet trajectories include Group 1, Group 3, and Group 4. Group 1 was characterized by an unhealthy diet score at baseline, with sustained improvement, while Group 3 showed a moderate diet score at baseline but persistent worsening, and Group 4 had healthy diet scores at baseline but persistent worsening. These groups of participants had a higher risk of adverse obesity and abdominal obesity than those participants who had healthy diet scores at baseline and sustained changes for the better.

LCD and LFD, a composite score encompassing the three primary macronutrient sources, can effectively unveil macronutrient intake and its respective sources. The association between LCD and LFD diets and obesity has been studied in national and international research but is not conclusive. In a nationwide cross-sectional study, researchers found that a healthy LCD was associated with decreased odds of steatosis, while an unhealthy LFD was linked to increased odds of steatosis (27). Findings from 37,233 US adults demonstrated that unhealthy LCD and LFD scores were linked to higher mortality, while healthy LCD and LFD scores were related to lower mortality (13). One study using data from NHANES found a significant association between insistence on healthy LCDs and LFDs and a lower risk of mortality among adults with prediabetes, while an unhealthy number of points for both LCDs and LFDs tended to be associated with a higher risk of mortality (43). In contrast, a cross-sectional study conducted in Iran failed to establish a correlation between low-carbohydrate diets and increased levels of overweight and obesity (20).

In addition to inconsistent associations, previous studies have relied on single point measurements, and there is a lack of data based on multiple point measurements. We add to this literature by using a group-based multitrajectory method, which clusters distinct trajectory patterns considering more than one variable and considering its association with the trajectory of adiposity. We documented how the long-term trends between these scores are important to fully understand the quality and quantity of an individual's macronutrient intake, which can incorporate the intercorrelations between LCDs and LFDs and enhance the precision of personalized group membership probabilities. In addition, the state of obesity is not a static condition, but rather a dynamic process of change. We used repeated measures of overweight upwards trajectories covering more than 15 years rather than single-point measurements of overweight upwards trajectories to facilitate a comprehensive consideration of the dynamics. This strategy can describe in a straightforward manner how BMI and WHR may increase, decrease, or remain stable in different groups with varying initial BMI and WHR values. By mapping the BMI or WHR trajectories, it is intuitive to identify specific groups.

However, it is important to note that the association between slow growth of abdominal obesity and adverse dietary trajectories was not statistically significant and there was even a negative association in one diet trajectory. In contrast to other studies of a similar nature, this study reached inconsistent conclusions with other studies due to differences in assessment methodology, as few studies have examined dual trajectory associations (9, 44). The trajectory of slow growth of abdominal obesity was non-abdominal in the first few years of follow-up and did not become abdominal obesity until the last 2 years of follow-up when most of the participants had reached middle age. Obesity results from an imbalance between energy intake and energy expenditure. The gradual loss of muscle mass that occurs with age can potentially affect habitual energy expenditure and the energy imbalance that leads to the development of obesity (45). In addition, middle-aged and older adults have a lower metabolic rate than adolescents (46). As a result, middle-aged and older adults are at greater risk of obesity than their younger counterparts with an equivalent dietary intake. Obesity due to age-related metabolic decline may explain the lack of statistical significance of the association between poor dietary habits and slow-growing abdominal obesity.

Several possible mechanisms may clarify the association between unhealthy LCD and LFD scores with adiposity. Saturated fats have a high energy density, and may directly affect energy expenditure and storage. This further leads to an increased risk of adiposity and abdominal obesity. LCDs, such as refined grains and added sugars, could be associated with adiposity and dyslipidemia because of their high glycemic index (47, 48). High-quality carbohydrates, which include non-starchy vegetables, whole fruits, and whole grains, are rich in fiber and can slow glucose absorption (49). High-fiber diets give the feeling of fullness without overloading us with calories, which could lower the risk of the population being overweight or obese. Based on the given explanation, we can shed light on the lower risk of healthy LCD and LFD scores and the trajectories of adverse adiposity.

The main strengths of this study include repeating measures of BMI and WHR and a long follow-up and various dimensions for diet scores allowing the assessment of dietary intake use dynamic changes. Based on this nationally representative sample of Chinese adults, we mapped the trajectory of change in diet and adiposity which can reflect Chinese dietary intake and adiposity status from 1997 to 2011. Moreover, this study provides insight into the association between dietary intake and adiposity trends. We also undertook inverse-probability weighting and multiple imputations in the sensitivity analysis phase, which takes missing data into account, and the results from these analyses were consistent with the main findings. Our study also had some limitations. First, although the 24-h dietary review method is a frequently used dietary evaluation tool, self-reported dietary information is subject to potential recall bias. Second, this study cannot preclude residual confounding despite our adjustment for many covariates. Third, it is important to emphasize that our assessment of obesity was based on BMI and WHR, while other tools for assessing obesity, such as anthropometric indices, were not included in this study. However, it is undeniable that BMI and WHR are currently the more commonly used indicators to assess obesity (50). Further studies need to objectively evaluate dietary intake to confirm our findings, and more experimental studies are needed to explain the mechanism.

## 5 Conclusion

Our findings, based on a nationally representative longitudinal survey, identified four distinct multitrajectories of LCD and LFD scores and concluded that maintaining healthy LCDs and LFDs can markedly decrease the risk of adiposity. The findings underscore the necessity for population-representative research on the trajectory of dietary patterns, which can establish an evidentiary basis for the development of guidelines, policies, and intervention targets aimed at sustaining healthy food environments.

## Data availability statement

The original contributions presented in the study are included in the article/Supplementary material, further inquiries can be directed to the corresponding authors: Siying Wu, Huangyuan Li, and Xiaoxu Xie.

## Ethics statement

The study was conducted according to the guidelines of the Declaration of Helsinki and was approved by the Institutional Review Board of the University of North Carolina at Chapel Hill, Chapel Hill, North Carolina, United States (No. 07-1963). The National Institute for Nutrition and Health, Chinese Center for Disease Control and Prevention, approved the survey (No. 201524). All subjects involved in the study gave their written informed consent.

## Author contributions

LZ and JG designed the research programs. LZ, XL, and XXu contributed to the data analysis. XL, XXu, and LY drafted the manuscript. SW, HL, and XXi reviewed the manuscript. All authors were involved in writing the paper and had final approval of the submitted and published versions and willing to share the study protocol and statistical code to generate the published results and data set with the journal editors.
