# Supplementary material for: Association between longitudinal dietary patterns and changes in obesity: a population-based cohort study
Source: Front Public Health. 2023 Dec 1;11:1227994. doi: 10.3389/fpubh.2023.1227994 (PMC10722423; doi:10.3389/fpubh.2023.1227994)
Supplement: Supplementary file 1 [file Data_Sheet_1.docx]

**Supplementary Material**

**Article title**

Association between longitudinal dietary patterns and changes in obesity: A population-based cohort study

**Author names**

Liuyan Zheng^1^**^†^**, Xinyue Lu^1^**^†^,** Jianhui Guo^1^**^†^**, Xingyan Xu^1^, Le Yang^1^, Xiaoxu Xie^1^**^*^**, Huangyuan Li^2^**^*^,** Siying Wu^1^**^*^**

**^†^**These authors contributed equally as co-first authors for this article.

**Authors’ affiliations**

^1^Department of Epidemiology and Health Statistics, School of Public Health, Fujian Medical University, Fujian, China

^2^Department of Preventive Medicine, School of Public Health, Fujian Medical University, Fujian, China

**Affiliation and e-mail address of the corresponding author**

Siying Wu, PhD, Department of Epidemiology and Health Statistics, School of Public Health, Fujian Medical University, 1 Xuefu Road, Fuzhou 350122, Fujian, China; E-mail: [fmuwsy@163.com](mailto:fmuwsy@163.com;); Telephone/Fax: +86-13305025606

Huangyuan Li, Ph.D, Department of Preventive Medicine, School of Public Health, Fujian Medical University, 1 Xuefu Road, Fuzhou 350122, Fujian, China; E-mail: fmulhy@163.com ; Telephone/Fax: +86-13067406829

Xiaoxu Xie, Ph.D, Department of Epidemiology and Health Statistics, School of Public Health, Fujian Medical University, 1 Xuefu Road, Fuzhou 350122, Fujian, China; E-mail: [xiexiaoxu@aliyun.com](mailto:xiexiaoxu@aliyun.com); Telephone/Fax: +86 0591 2286 2023

**Appendix A. Supplementary data**

**Association between longitudinal dietary patterns and changes in obesity: A population-based cohort study**

**Figure S1.** Flowchart of the participants included in the current analysis.

**Table S1.** Criteria for determining the low-carbohydrate diet (LCD) scores

**Table S2.** Criteria for determining the low-fat diet (LFD) scores

**Table S3.** Distribution of energy percentage criteria for determining scores for low-carbohydrate and low-fat diets in men

**Table S4.** Distribution of energy percentage criteria for determining scores for low-carbohydrate and low-fat diets in women

**Table S5.** The association between the obesity trajectory and the change trajectory patterns of low-carbohydrate and low-fat diet scores using the propensity scores of IPTW

**Table S6.** The associations between the abdominal obesity trajectory and the change trajectory patterns of low-carbohydrate and low-fat diet scores using the propensity scores of IPTW

**Table S7.** The association between the obesity trajectory and the change trajectory patterns of low-carbohydrate and low-fat diet scores using multiple imputation approach

**Table S8.** The associations between the abdominal obesity trajectory and the change trajectory patterns of low-carbohydrate and low-fat diet scores using multiple imputation approach

**Table S9.** The associations between the obesity trajectory and the change trajectory patterns of low-carbohydrate and low-fat diet scores by excluding participants with diabetes at baseline

**Table S10.** The associations between the abdominal obesity trajectory and the change trajectory patterns of low-carbohydrate and low-fat diet scores by excluding participants with diabetes at baseline

**Table S11.** The associations between the obesity trajectory and the change trajectory patterns of low-carbohydrate and low-fat diet scores by excluding participants with diabetes at baseline and at all follow-up years

**Table S12.** The associations between the abdominal obesity trajectory and the change trajectory patterns of low-carbohydrate and low-fat diet scores by excluding participants with diabetes at baseline and at all follow-up years


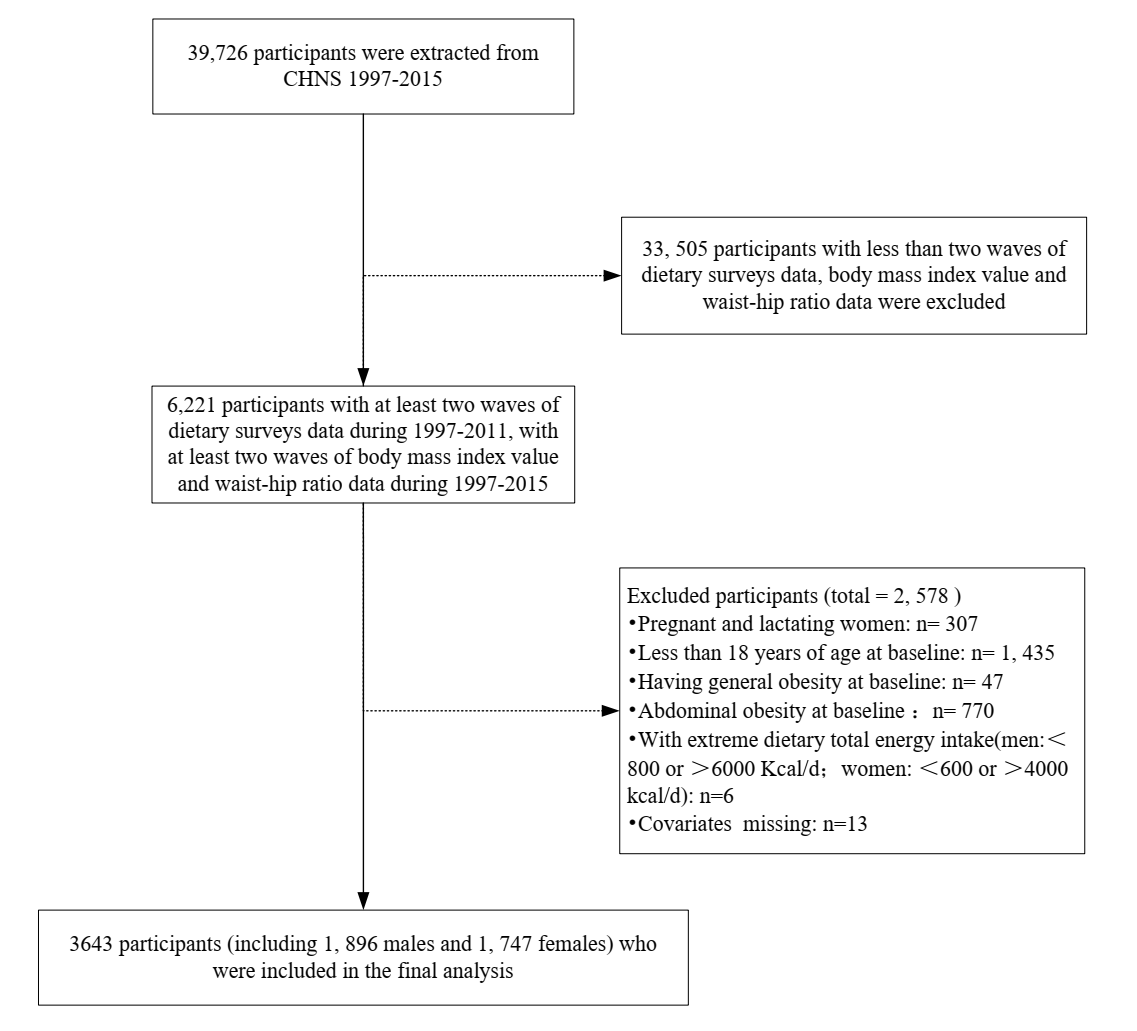


**Figure S1.** Flowchart of the participants included in the current analysis.

**Table S1.** Criteria for determining the low-carbohydrate diet (LCD) scores

| Points | Healthful LCD score | | | Unhealthful LCD score | | |
| --- | --- | --- | --- | --- | --- | --- |
|  | Low-quality carbohydrates | Unsaturated  fats | Plant  proteins | High-quality carbohydrates | Saturated fats | Animal  proteins |
| 0 | RS | PS | PS | RS | PS | PS |
| 1 |  |  |  |  |  |  |
| 2 |  |  |  |  |  |  |
| 3 |  |  |  |  |  |  |
| 4 |  |  |  |  |  |  |
| 5 |  |  |  |  |  |  |
| 6 |  |  |  |  |  |  |
| 7 |  |  |  |  |  |  |
| 8 |  |  |  |  |  |  |
| 9 |  |  |  |  |  |  |
| 10 |  |  |  |  |  |  |

Abbreviations: LCD, Low-carbohydrate diet; RS, Reverse scores; PS, Positive scores.

RS: Scores from 10 (the lowest stratum) to 0 (the highest stratum).

PS: Scores from 0 (the lowest stratum) to 10 (the highest stratum).

High-quality carbohydrates: Carbohydrates from whole grains, whole fruit, legumes, and non-starchy vegetables.

Low-quality carbohydrates: Carbohydrates from refined grains, added sugar, fruit juice, potato, and other starchy vegetables.

Unsaturated fatty acids: Monounsaturated fats and polyunsaturated fats.

Plant proteins: Proteins from whole grains, refined grains, nuts, legumes, soy, and other plant sources.

Animal proteins: Proteins from fish/seafood, unprocessed red meat, processed meat, poultry, egg, dairy products, and other animal sources.

**Table S2.** Criteria for determining the low-fat diet (LFD) scores

| Points | Healthful LFD score | | | Unhealthful LFD score | | |
| --- | --- | --- | --- | --- | --- | --- |
|  | Saturated fats | High-quality carbohydrates | Plant proteins | Unsaturated fats | Low-quality carbohydrates | Animal proteins |
| 0 | RS | PS | PS | RS | PS | PS |
| 1 |  |  |  |  |  |  |
| 2 |  |  |  |  |  |  |
| 3 |  |  |  |  |  |  |
| 4 |  |  |  |  |  |  |
| 5 |  |  |  |  |  |  |
| 6 |  |  |  |  |  |  |
| 7 |  |  |  |  |  |  |
| 8 |  |  |  |  |  |  |
| 9 |  |  |  |  |  |  |
| 10 |  |  |  |  |  |  |

Abbreviations: LFD, Low-fat diet; RS, Reverse scores; PS, Positive scores.

RS: Scores from 10 (the lowest stratum) to 0 (the highest stratum).

PS: Scores from 0 (the lowest stratum) to 10 (the highest stratum).

High-quality carbohydrates: Carbohydrates from whole grains, whole fruit, legumes, and non-starchy vegetables.

Low-quality carbohydrates: Carbohydrates from refined grains, added sugar, fruit juice, potato, and other starchy vegetables.

Unsaturated fatty acids: Monounsaturated fats and polyunsaturated fats.

Plant proteins: Proteins from whole grains, refined grains, nuts, legumes, soy, and other plant sources.

Animal proteins: Proteins from fish/seafood, unprocessed red meat, processed meat, poultry, egg, dairy products, and other animal sources.

**Table S3.** Distribution of energy percentage criteria for determining scores for low-carbohydrate and low-fat diets in men

| Variables | | Sustained healthy  dietary habits | Improved dietary habits | Worsening dietary habits | Deteriorating dietary habits |
| --- | --- | --- | --- | --- | --- |
|  |  | 1997 (Percentage of energy) | | | |
| ULCD | High-quality carbohydrate | 2.16(1.02, 4.22) | 2.35 (1.22, 5.88) | 2.08(1.30, 4.44) | 2.21(1.28, 4.80) |
|  | Animal protein | 0.59(0.00, 1.73) | 3.77(2.35, 6.18) | 5.13(2.70, 8.09) | 0.65(0.00, 1.59) |
|  | Saturated fat | 0.00(0.00,1.78) | 8.87(4.75,13.41) | 13.79(8.94, 20.59) | 0.00(0.00, 1.68) |
| HLCD | Low-quality carbohydrate | 78.98(71.36, 82.77) | 60.07(50.70, 68.99) | 60.09(46.66, 69.82) | 77.29(71.31, 82.22) |
|  | Plant protein | 10.97(10.14, 11.96) | 10.07(8.71, 11.80) | 8.81(7.79, 9.99) | 11.07(10.22, 12.29) |
|  | Unsaturated fat | 4.69(3.67, 6.37) | 7.85(5.55, 11.97) | 5.31(3.24, 8.74) | 4.74(3.56, 6.69) |
| ULFD | Unsaturated fat | 4.69(3.67, 6.37) | 7.85(5.55, 11.97) | 5.31(3.24, 8.74) | 4.74(3.56, 6.69) |
|  | Low-quality carbohydrate | 78.98(71.36, 82.77) | 60.07(50.70, 68.99) | 60.09(46.66, 69.82) | 77.29(71.31, 82.22) |
|  | Animal protein | 0.59(0.00, 1.73) | 3.77(2.35, 6.18) | 5.13(2.70, 8.09) | 0.65(0.00, 1.59) |
| HLFD | Saturated fat | 0.00(0.00,1.78) | 8.87(4.75,13.41) | 13.79(8.94, 20.59) | 0.00(0.00, 1.68) |
|  | High-quality carbohydrate | 2.16(1.02, 4.22) | 2.35 (1.22, 5.88) | 2.08(1.30, 4.44) | 2.21(1.28, 4.80) |
|  | Plant protein | 10.97(10.14, 11.96) | 10.07(8.71, 11.80) | 8.81(7.79, 9.99) | 11.07(10.22, 12.29) |
|  |  | 2000 (Percentage of energy) | | | |
| ULCD | High-quality carbohydrate | 2.51(1.40, 5.57) | 2.54(1.37, 5.31) | 2.05(1.23, 4.25) | 2.14(1.30, 5.48) |
|  | Animal protein | 1.25(0.00, 2.74) | 4.45(2.81, 6.65) | 5.31(3.81, 7.55) | 1.60(0.18, 3.24) |
|  | Saturated fat | 0.00(0.00, 6.51) | 10.78(5.40, 17.31) | 14.69(8.25, 20.74) | 0.00(0.00, 5.43) |
| HLCD | Low-quality carbohydrate | 72.88(63.89, 79.16) | 56.51(47.34, 65.82) | 59.92(52.69, 67.06) | 75.37(67.40, 79.98) |
|  | Plant protein | 11.17(10.03, 12.49) | 9.57(8.35, 11.05) | 8.53(7.51, 9.59) | 10.78(9.50, 12.03) |
|  | Unsaturated fat | 5.29(3.88, 7.67) | 8.34(5.52, 12.20) | 5.33(3.93, 7.87) | 4.72(3.40, 6.46) |
| ULFD | Unsaturated fat | 5.29(3.88, 7.67) | 8.34(5.52, 12.20) | 5.33(3.93, 7.87) | 4.72(3.40, 6.46) |
|  | Low-quality carbohydrate | 72.88(63.89, 79.16) | 56.51(47.34, 65.82) | 59.92(52.69, 67.06) | 75.37(67.40, 79.98) |
|  | Animal protein | 1.25(0.00, 2.74) | 4.45(2.81, 6.65) | 5.31(3.81, 7.55) | 1.60(0.18, 3.24) |
| HLFD | Saturated fat | 0.00(0.00, 6.51) | 10.78(5.40, 17.31) | 14.69(8.25, 20.74) | 0.00(0.00, 5.43) |
|  | High-quality carbohydrate | 2.51(1.40, 5.57) | 2.54(1.37, 5.31) | 2.05(1.23, 4.25) | 2.14(1.30, 5.48) |
|  | Plant protein | 11.17(10.03, 12.49) | 9.57(8.35, 11.05) | 8.53(7.51, 9.59) | 10.78(9.50, 12.03) |
|  |  | 2004 (Percentage of energy) | | | |
| ULCD | High-quality carbohydrate | 41.81(18.44, 58.31) | 21.46(7.76, 42.49) | 7.18(3.52,24.18) | 23.06(5.61, 47.96) |
|  | Animal protein | 1.17(0.00, 2.81) | 4.50(2.86, 6.68) | 6.60(4.83, 8.73) | 2.36(1.03, 4.31) |
|  | Saturated fat | 0.00(0.00, 6.06) | 10.12(5.48, 15.06) | 15.42(8.83, 21.16) | 2.88(0.00, 9.47) |
| HLCD | Low-quality carbohydrate | 34.76(18.59, 59.35) | 37.62(20.44, 53.55) | 48.64(29.08, 60.70) | 49.54(25.51, 69.01) |
|  | Plant protein | 10.58(9.53, 11.62) | 9.86(8.59, 11.22) | 8.37(7.38, 9.70) | 10.34(8.93, 11.62) |
|  | Unsaturated fat | 5.49(3.64, 11.52) | 10.14(6.50, 16.79) | 6.54(4.12, 10.34) | 5.32(3.64, 8.85) |
| ULFD | Unsaturated fat | 5.49(3.64, 11.52) | 10.14(6.50, 16.79) | 6.54(4.12, 10.34) | 5.32(3.64, 8.85) |
|  | Low-quality carbohydrate | 34.76(18.59, 59.35) | 37.62(20.44, 53.55) | 48.64(29.08, 60.70) | 49.54(25.51, 69.01) |
|  | Animal protein | 1.17(0.00, 2.81) | 4.50(2.86, 6.68) | 6.60(4.83, 8.73) | 2.36(1.03, 4.31) |
| HLFD | Saturated fat | 0.00(0.00, 6.06) | 10.12(5.48, 15.06) | 15.42(8.83, 21.16) | 2.88(0.00, 9.47) |
|  | High-quality carbohydrate | 41.81(18.44, 58.31) | 21.46(7.76, 42.49) | 7.18(3.52,24.18) | 23.06(5.61, 47.96) |
|  | Plant protein | 10.58(9.53, 11.62) | 9.86(8.59, 11.22) | 8.37(7.38, 9.70) | 10.34(8.93, 11.62) |
|  |  | 2006 (Percentage of energy) | | | |
| ULCD | High-quality carbohydrate | 45.64(28.97, 59.73) | 26.16(11.89, 42.59) | 15.50(4.77, 35.46) | 24.40(7.05, 48.54) |
|  | Animal protein | 1.17(0.00, 2.81) | 4.50(2.86, 6.68) | 6.60(4.83, 8.73) | 2.35(1.03, 4.31) |
|  | Saturated fat | 2.37(0.00, 7.74) | 10.65(5.60, 15.84) | 16.16(10.38, 21.67) | 6.62(0.00, 13.04) |
| HLCD | Low-quality carbohydrate | 34.76(18.59, 59.35) | 37.62(20.44, 53.55) | 48.64(29.08, 60.70) | 49.54(25.51, 69.01) |
|  | Plant protein | 10.49(9.35, 11.88) | 9.83(8.46, 11.40) | 8.68(7.34, 9.90) | 10.15(8.91, 11.72) |
|  | Unsaturated fat | 7.35(4.05, 16.18) | 11.48(7.70, 17.61) | 7.70(5.07,10.78) | 6.77(4.14, 10.37) |
| ULFD | Unsaturated fat | 7.35(4.05, 16.18) | 11.48(7.70, 17.61) | 7.70(5.07,10.78) | 6.77(4.14, 10.37) |
|  | Low-quality carbohydrate | 34.76(18.59, 59.35) | 37.62(20.44, 53.55) | 48.64(29.08, 60.70) | 49.54(25.51, 69.01) |
|  | Animal protein | 1.17(0.00, 2.81) | 4.50(2.86, 6.68) | 6.60(4.83, 8.73) | 2.35(1.03, 4.31) |
| HLFD | Saturated fat | 2.37(0.00, 7.74) | 10.65(5.60, 15.84) | 16.16(10.38, 21.67) | 6.62(0.00, 13.04) |
|  | High-quality carbohydrate | 45.64(28.97, 59.73) | 26.16(11.89, 42.59) | 15.50(4.77, 35.46) | 24.40(7.05, 48.54) |
|  | Plant protein | 10.49(9.35, 11.88) | 9.83(8.46, 11.40) | 8.68(7.34, 9.90) | 10.15(8.91, 11.72) |
|  |  | 2009 (Percentage of energy) | | | |
| ULCD | High-quality carbohydrate | 43.72(29.21, 57.29) | 25.91(9.66, 44.27) | 14.26(5.80, 37.32) | 21.69(7.80, 38.92) |
|  | Animal protein | 2.43(1.13, 3.82) | 4.59(3.13, 6.43) | 6.94(5.08, 8.81) | 3.67(2.17, 5.69) |
|  | Saturated fat | 2.89(0.00, 8.46) | 10.13(5.77, 15.14) | 15.77(10.93, 21.25) | 8.27(2.57, 14.89) |
| HLCD | Low-quality carbohydrate | 27.19(15.45,41.65) | 34.60(19.30, 49.58) | 37.61(20.68, 50.50) | 49.44(27.88, 61.02) |
|  | Plant protein | 10.34(9.21, 11.93) | 9.83(8.68, 11.13) | 8.30(7.37, 9.60) | 9.61(8.44, 10.91) |
|  | Unsaturated fat | 8.18(5.13,13.67) | 11.71(7.56, 18.81) | 7.92(5.19, 12.01) | 6.43(4.32, 9.92) |
| ULFD | Unsaturated fat | 8.18(5.13,13.67) | 11.71(7.56, 18.81) | 7.92(5.19, 12.01) | 6.43(4.32, 9.92) |
|  | Low-quality carbohydrate | 27.19(15.45,41.65) | 34.60(19.30, 49.58) | 37.61(20.68, 50.50) | 49.44(27.88, 61.02) |
|  | Animal protein | 2.43(1.13, 3.82) | 4.59(3.13, 6.43) | 6.94(5.08, 8.81) | 3.67(2.17, 5.69) |
| HLFD | Saturated fat | 2.89(0.00, 8.46) | 10.13(5.77, 15.14) | 15.77(10.93, 21.25) | 8.27(2.57, 14.89) |
|  | High-quality carbohydrate | 43.72(29.21, 57.29) | 25.91(9.66, 44.27) | 14.26(5.80, 37.32) | 21.69(7.80, 38.92) |
|  | Plant protein | 10.34(9.21, 11.93) | 9.83(8.68, 11.13) | 8.30(7.37, 9.60) | 9.61(8.44, 10.91) |
|  |  | 2011 (Percentage of energy) | | | |
| ULCD | High-quality carbohydrate | 26.11(12.47, 47.06) | 11.39(6.67, 21.83) | 6.58(3.89, 11.14) | 5.98(3.89, 11.82) |
|  | Animal protein | 2.52(1.24, 4.06) | 4.94(3.22, 7.43) | 6.89(4.99, 9.63) | 4.19(2.48, 6.18) |
|  | Saturated fat | 3.86(0.00, 6.73) | 10.08(5.70, 15.09) | 15.59(9.78, 22.50) | 9.30(4.25, 14.66) |
| HLCD | Low-quality carbohydrate | 42.88(22.62, 56.24) | 45.67(34.99, 56.39) | 50.44(40.49, 59.05) | 62.13(52.37, 68.83) |
|  | Plant protein | 11.05(9.80, 12.98) | 10.09(8.64, 11.66) | 8.89(7.43, 10.23) | 9.94(8.77, 11.09) |
|  | Unsaturated fat | 9.00(5.52, 13.19) | 11.56(8.43, 15.86) | 8.47(5.62, 12.08) | 6.65(4.87, 9.76) |
| ULFD | Unsaturated fat | 9.00(5.52, 13.19) | 11.56(8.43, 15.86) | 8.47(5.62, 12.08) | 6.65(4.87, 9.76) |
|  | Low-quality carbohydrate | 42.88(22.62, 56.24) | 45.67(34.99, 56.39) | 50.44(40.49, 59.05) | 62.13(52.37, 68.83) |
|  | Animal protein | 2.52(1.24, 4.06) | 4.94(3.22, 7.43) | 6.89(4.99, 9.63) | 4.19(2.48, 6.18) |
| HLFD | Saturated fat | 3.86(0.00, 6.73) | 10.08(5.70, 15.09) | 15.59(9.78, 22.50) | 9.30(4.25, 14.66) |
|  | High-quality carbohydrate | 26.11(12.47, 47.06) | 11.39(6.67, 21.83) | 6.58(3.89, 11.14) | 5.98(3.89, 11.82) |
|  | Plant protein | 11.05(9.80, 12.98) | 10.09(8.64, 11.66) | 8.89(7.43, 10.23) | 9.94(8.77, 11.09) |

variables were presented as P_50_ (P_25_, P_75_).

**Table S4.** Distribution of energy percentage criteria for determining scores for low-carbohydrate and low-fat diets in women

| Variables | | Sustained healthy  dietary habits | Improved  dietary habits | Worsening dietary habits | Deteriorating dietary habits |
| --- | --- | --- | --- | --- | --- |
|  |  | 1997 (Percentage of energy) | | | |
| ULCD | High-quality carbohydrate | 1.86(1.20, 2.84) | 1.85(0.91, 2.98) | 4.36(1.91,10.96) | 3.11(1.70, 7.29) |
|  | Animal protein | 0.51(0.00, 1.45) | 3.81(2.13, 6.12) | 1.73(0.43, 3.65) | 6.26(2.77, 8.96) |
|  | Saturated fat | 0.00(0.00, 0.94) | 11.18(5.27, 17.03) | 1.84(0.00, 7.19) | 10.13(5.65, 16.27) |
| HLCD | Low-quality carbohydrate | 68.39(56.17,78.04) | 67.17(59.93, 74.08) | 65.81(52.05, 76.96) | 58.75(45.93, 69.96) |
|  | Plant protein | 10.79(10.07,11.58) | 8.95(8.07, 10.18) | 11.95(10.67, 13.28) | 9.61(8.55, 10.80) |
|  | Unsaturated fat | 4.37(3.35,5.96) | 5.48(3.67,7.75) | 7.56(5.64, 12.14) | 7.52(4.90, 11.88) |
| ULFD | Unsaturated fat | 4.37(3.35,5.96) | 5.48(3.67,7.75) | 7.56(5.64, 12.14) | 7.52(4.90, 11.88) |
|  | Low-quality carbohydrate | 68.39(56.17,78.04) | 67.17(59.93, 74.08) | 65.81(52.05, 76.96) | 58.75(45.93, 69.96) |
|  | Animal protein | 0.51(0.00, 1.45) | 3.81(2.13, 6.12) | 1.73(0.43, 3.65) | 6.26(2.77, 8.96) |
| HLFD | Saturated fat | 0.00(0.00, 0.94) | 11.18(5.27, 17.03) | 1.84(0.00, 7.19) | 10.13(5.65, 16.27) |
|  | High-quality carbohydrate | 1.86(1.20, 2.84) | 1.85(0.91, 2.98) | 4.36(1.91,10.96) | 3.11(1.70, 7.29) |
|  | Plant protein | 10.79(10.07,11.58) | 8.95(8.07, 10.18) | 11.95(10.67, 13.28) | 9.61(8.55, 10.80) |
|  |  | 2000 (Percentage of energy) | | | |
| ULCD | High-quality carbohydrate | 2.38(1.35, 5.20) | 2.08(1.09, 3.91) | 3.94(2.00, 8.88) | 2.99(1.62, 6.50) |
|  | Animal protein | 1.29(0.00, 2.99) | 4.74(2.98, 6.57) | 2.51(0.94, 4.62) | 5.46(3.35, 7.55) |
|  | Saturated fat | 0.00(0.00, 6.33) | 11.11(4.72, 17.50) | 3.33(0.00, 9.70) | 12.23(6.48, 18.07) |
| HLCD | Low-quality carbohydrate | 74.17(65.41, 80.30) | 63.69(55.64, 72.15) | 63.72(51.96, 72.60) | 60.34(49.61, 67.30) |
|  | Plant protein | 10.93(9.77, 12.22) | 8.89(7.86, 10.18) | 11.42(10.03, 12.88) | 9.32(8.03, 10.45) |
|  | Unsaturated fat | 5.02(3.44, 7.51) | 5.63(3.80, 8.66) | 8.83(6.16, 13.76) | 7.20(4.91, 9.37) |
| ULFD | Unsaturated fat | 5.02(3.44, 7.51) | 5.63(3.80, 8.66) | 8.83(6.16, 13.76) | 7.20(4.91, 9.37) |
|  | Low-quality carbohydrate | 74.17(65.41, 80.30) | 63.69(55.64, 72.15) | 63.72(51.96, 72.60) | 60.34(49.61, 67.30) |
|  | Animal protein | 1.29(0.00, 2.99) | 4.74(2.98, 6.57) | 2.51(0.94, 4.62) | 5.46(3.35, 7.55) |
| HLFD | Saturated fat | 0.00(0.00, 6.33) | 11.11(4.72, 17.50) | 3.33(0.00, 9.70) | 12.23(6.48, 18.07) |
|  | High-quality carbohydrate | 2.38(1.35, 5.20) | 2.08(1.09, 3.91) | 3.94(2.00, 8.88) | 2.99(1.62, 6.50) |
|  | Plant protein | 10.93(9.77, 12.22) | 8.89(7.86, 10.18) | 11.42(10.03, 12.88) | 9.32(8.03, 10.45) |
|  |  | 2004 (Percentage of energy) | | | |
| ULCD | High-quality carbohydrate | 40.24(18.37, 58.09) | 24.89(7.11, 43.72) | 29.52(9.10, 53.76) | 9.51(4.63, 27.35) |
|  | Animal protein | 1.12(0.00, 2.48) | 4.49(2.86, 6.59) | 3.19(1.57, 5.07) | 6.11(3.74, 8.64) |
|  | Saturated fat | 0.00(0.00, 6.08) | 10.46(4.64, 16.53) | 4.40(0.00, 10.43) | 13.11(7.39, 18.74) |
| HLCD | Low-quality carbohydrate | 37.90(22.46, 62.32) | 39.43(23.01, 60.46) | 41.59(20.11, 59.35) | 49.40(32.45, 60.38) |
|  | Plant protein | 10.92(9.95, 12.26) | 9.64(8.12, 10.77) | 10.57(9.47, 12.22) | 8.97(7.67, 10.31) |
|  | Unsaturated fat | 5.45(3.63, 9.82) | 7.63(4.89, 12.90) | 9.74(5.67, 17.37) | 7.03(4.70, 10.85) |
| ULFD | Unsaturated fat | 5.45(3.63, 9.82) | 7.63(4.89, 12.90) | 9.74(5.67, 17.37) | 7.03(4.70, 10.85) |
|  | Low-quality carbohydrate | 37.90(22.46, 62.32) | 39.43(23.01, 60.46) | 41.59(20.11, 59.35) | 49.40(32.45, 60.38) |
|  | Animal protein | 1.12(0.00, 2.48) | 4.49(2.86, 6.59) | 3.19(1.57, 5.07) | 6.11(3.74, 8.64) |
| HLFD | Saturated fat | 0.00(0.00, 6.08) | 10.46(4.64, 16.53) | 4.40(0.00, 10.43) | 13.11(7.39, 18.74) |
|  | High-quality carbohydrate | 40.24(18.37, 58.09) | 24.89(7.11, 43.72) | 29.52(9.10, 53.76) | 9.51(4.63, 27.35) |
|  | Plant protein | 10.92(9.95, 12.26) | 9.64(8.12, 10.77) | 10.57(9.47, 12.22) | 8.97(7.67, 10.31) |
|  |  | 2006 (Percentage of energy) | | | |
| ULCD | High-quality carbohydrate | 46.76(28.81, 62.59) | 29.71(10.83, 45.21) | 35.59(14.05, 49.78) | 17.50(6.34, 35.17) |
|  | Animal protein | 1.69(0.59, 3.29) | 4.78(3.47, 6.65) | 3.52(2.19, 5.56) | 6.87(4.66, 9.02) |
|  | Saturated fat | 1.85(0.00, 6.93) | 11.16(5.46, 16.19) | 8.32(2.45, 13.28) | 16.06(9.09, 22.53) |
| HLCD | Low-quality carbohydrate | 29.15(15.70, 46.13) | 34.19(18.99, 50.73) | 33.28(19.46, 51.17) | 41.76(21.43, 52.95) |
|  | Plant protein | 10.90(9.54, 12.19) | 9.81(8.56, 11.28) | 10.41(9.10, 12.17) | 8.75(7.52, 10.27) |
|  | Unsaturated fat | 7.13(4.26,15.78) | 10.36(6.21, 16.03) | 9.97(5.96, 18.29) | 8.15(5.23, 11.49) |
| ULFD | Unsaturated fat | 7.13(4.26,15.78) | 10.36(6.21, 16.03) | 9.97(5.96, 18.29) | 8.15(5.23, 11.49) |
|  | Low-quality carbohydrate | 29.15(15.70, 46.13) | 34.19(18.99, 50.73) | 33.28(19.46, 51.17) | 41.76(21.43, 52.95) |
|  | Animal protein | 1.69(0.59, 3.29) | 4.78(3.47, 6.65) | 3.52(2.19, 5.56) | 6.87(4.66, 9.02) |
| HLFD | Saturated fat | 1.85(0.00, 6.93) | 11.16(5.46, 16.19) | 8.32(2.45, 13.28) | 16.06(9.09, 22.53) |
|  | High-quality carbohydrate | 46.76(28.81, 62.59) | 29.71(10.83, 45.21) | 35.59(14.05, 49.78) | 17.50(6.34, 35.17) |
|  | Plant protein | 10.90(9.54, 12.19) | 9.81(8.56, 11.28) | 10.41(9.10, 12.17) | 8.75(7.52, 10.27) |
|  |  | 2009 (Percentage of energy) | | | |
| ULCD | High-quality carbohydrate | 46.88(35.91, 60.49) | 33.79(17.47, 50.84) | 22.99(9.08, 44.54) | 14.18(7.41, 33.17) |
|  | Animal protein | 2.26(1.02, 3.77) | 4.67(3.14, 6.54) | 4.35(2.71, 6.15) | 6.63(4.84, 8.80) |
|  | Saturated fat | 1.88(0.00, 7.83) | 10.19(5.79, 14.76) | 8.45(3.73, 13.68) | 15.61(10.48, 21.01) |
| HLCD | Low-quality carbohydrate | 26.3(16.14, 41.06) | 31.19(16.50, 45.85) | 43.96(25.52, 57.04) | 42.01(27.84, 53.07) |
|  | Plant protein | 10.57(9.52, 11.98) | 9.94(8.74, 11.55) | 10.13(8.97, 11.53) | 8.70(7.47, 9.83) |
|  | Unsaturated fat | 7.76(5.11, 13.56) | 10.04(6.87, 15.37) | 9.39(6.07, 16.76) | 8.01(5.50, 12.28) |
| ULFD | Unsaturated fat | 7.76(5.11, 13.56) | 10.04(6.87, 15.37) | 9.39(6.07, 16.76) | 8.01(5.50, 12.28) |
|  | Low-quality carbohydrate | 26.3(16.14, 41.06) | 31.19(16.50, 45.85) | 43.96(25.52, 57.04) | 42.01(27.84, 53.07) |
|  | Animal protein | 2.26(1.02, 3.77) | 4.67(3.14, 6.54) | 4.35(2.71, 6.15) | 6.63(4.84, 8.80) |
| HLFD | Saturated fat | 1.88(0.00, 7.83) | 10.19(5.79, 14.76) | 8.45(3.73, 13.68) | 15.61(10.48, 21.01) |
|  | High-quality carbohydrate | 46.88(35.91, 60.49) | 33.79(17.47, 50.84) | 22.99(9.08, 44.54) | 14.18(7.41, 33.17) |
|  | Plant protein | 10.57(9.52, 11.98) | 9.94(8.74, 11.55) | 10.13(8.97, 11.53) | 8.70(7.47, 9.83) |
|  |  | 2011 (Percentage of energy) | | | |
| ULCD | High-quality carbohydrate | 34.65(19.55, 54.82) | 16.72(9.01, 32.49) | 9.55(5.04, 16.20) | 8.12(4.85,13.89) |
|  | Animal protein | 2.44(1.06, 3.77) | 4.42(2.70, 6.77) | 4.84(3.21, 7.32) | 7.15(4.99, 10.14) |
|  | Saturated fat | 3.11(0.00, 6.19) | 7.46(3.33, 11.99) | 10.07(5.89, 13.93) | 17.37(11.53, 24.24) |
| HLCD | Low-quality carbohydrate | 38.05(22.25, 50.98) | 44.42(33.46, 54.83) | 55.32(44.69, 65.23） | 49.27(39.55,58.20) |
|  | Plant protein | 11.39(10.18, 13.55) | 10.82(9.46, 12.24) | 10.08(8.91,11.21) | 8.87(7.60, 10.07) |
|  | Unsaturated fat | 8.82(5.80, 14.38) | 12.26(8.59, 17.08) | 9.09(6.30, 12.82) | 8.68(6.15,11.16) |
| ULFD | Unsaturated fat | 8.82(5.80, 14.38) | 12.26(8.59, 17.08) | 9.09(6.30, 12.82) | 8.68(6.15,11.16) |
|  | Low-quality carbohydrate | 38.05(22.25, 50.98) | 44.42(33.46, 54.83) | 55.32(44.69, 65.23） | 49.27(39.55,58.20) |
|  | Animal protein | 2.44(1.06, 3.77) | 4.42(2.70, 6.77) | 4.84(3.21, 7.32) | 7.15(4.99, 10.14) |
| HLFD | Saturated fat | 3.11(0.00, 6.19) | 7.46(3.33, 11.99) | 10.07(5.89, 13.93) | 17.37(11.53, 24.24) |
|  | High-quality carbohydrate | 34.65(19.55, 54.82) | 16.72(9.01, 32.49) | 9.55(5.04, 16.20) | 8.12(4.85,13.89) |
|  | Plant protein | 11.39(10.18, 13.55) | 10.82(9.46, 12.24) | 10.08(8.91,11.21) | 8.87(7.60, 10.07) |

variables were presented as P_50_ (P_25_, P_75_).

**Table S5.** The association between the obesity trajectory and the change trajectory patterns of low-carbohydrate and low-fat diet scores using the propensity scores of IPTW

| **Trajectories** | **Overweight trajectory vs normal weight trajectory** | |  | **Obesity trajectory vs normal weight trajectory** | |
| --- | --- | --- | --- | --- | --- |
|  | ***P* value** | ***OR* (95% *CI*)** |  | ***P* value** | ***OR* (95% *CI*)** |
| Group2, Healthy diet scores, sustained changed for the better | Reference | |  | Reference | |
| Group1, Unhealthy die score, sustained changed for the better | ＜0.001 | 1.703(1.417,2.046) |  | ＜0.001 | 1.559(1.274,1.907) |
| Group3, Moderate die score, persistent worsening | ＜0.001 | 1.446(1.192,1.754) |  | 0.007 | 1.313(1.076,1.602) |
| Group4, Healthy diet scores, persistent worsening | ＜0.001 | 1.372(1.145,1.644) |  | ＜0.001 | 1.702(1.403,2.065) |

Adjusted model: Adjusted for sociodemographic factors, including sex, age, marital status, nationality, education level, family economic level, region, and lifestyle factors, including smoking status, drinking status, physical activity, and dietary energy.

**Table S6.** The associations between the abdominal obesity trajectory and the change trajectory patterns of low-carbohydrate and low-fat diet scores using the propensity scores of IPTW

| **Trajectories** | **Slowly growth of abdominal obesity trajectory vs No**  **abdominal obesity group trajectory** | |  | **Rapidly growth of abdominal obesity trajectory vs No**  **abdominal obesity group trajectory** | |
| --- | --- | --- | --- | --- | --- |
|  | ***P* value** | ***OR* (95% *CI*)** |  | ***P* value** | ***OR* (95% *CI*)** |
| Group2, Healthy diet scores, sustained changed for the better | Reference | |  | Reference | |
| Group1, Unhealthy die score, sustained changed for the better | 0.985 | 1.002(0.847,1.185) |  | 0.002 | 1.311(1.108,1.551) |
| Group3, Moderate die score, persistent worsening | ＜0.001 | 0.737(0.620,0.877) |  | 0.064 | 1.179(0.991,1.404) |
| Group4, Healthy diet scores, persistent worsening | 0.008 | 1.242(1.057,1.459) |  | ＜0.001 | 1.356(1.150,1.600) |

Adjusted model: Adjusted for sociodemographic factors, including sex, age, marital status, nationality, education level, family economic level, region, and lifestyle factors, including smoking status, drinking status, physical activity, and dietary energy.

**Table S7.** The association between the obesity trajectory and the change trajectory patterns of low-carbohydrate and low-fat diet scores using multiple imputation approach

| **Trajectories** | **Overweight trajectory vs normal weight trajectory** | |  | **Obesity trajectory vs normal weight trajectory** | |
| --- | --- | --- | --- | --- | --- |
|  | ***P* value** | ***OR* (95% *CI*)** |  | ***P* value** | ***OR* (95% *CI*)** |
| Group2, Healthy diet scores, sustained changed for the better | Reference | |  | Reference | |
| Group1, Unhealthy die score, sustained changed for the better | ＜0.001 | 1.574(1.34,1.849) |  | ＜0.001 | 1.431(1.196,1.712) |
| Group3, Moderate die score, persistent worsening | ＜0.001 | 1.48(1.255,1.744) |  | 0.008 | 1.243(1.059,1.459) |
| Group4, Healthy diet scores, persistent worsening | 0.007 | 1.241(1.06,1.452) |  | ＜0.001 | 1.489(1.236,1.794) |

Adjusted model: Adjusted for sociodemographic factors, including sex, age, marital status, nationality, education level, family economic level, region, and lifestyle factors, including smoking status, drinking status, physical activity, and dietary energy.

**Table S8.** The associations between the abdominal obesity trajectory and the change trajectory patterns of low-carbohydrate and low-fat diet scores using multiple imputation approach

| **Trajectories** | **Slowly growth of abdominal obesity trajectory vs No**  **abdominal obesity group trajectory** | |  | **Rapidly growth of abdominal obesity trajectory vs No**  **abdominal obesity group trajectory** | |
| --- | --- | --- | --- | --- | --- |
|  | ***P* value** | ***OR* (95% *CI*)** |  | ***P* value** | ***OR* (95% *CI*)** |
| Group2, Healthy diet scores, sustained changed for the better | Reference | |  | Reference | |
| Group1, Unhealthy die score, sustained changed for the better | 0.193 | 0.895(0.757,1.058) |  | ＜0.001 | 1.339(1.141,1.571) |
| Group3, Moderate die score, persistent worsening | ＜0.001 | 0.722(0.614,0.850) |  | 0.011 | 1.231(1.049,1.445) |
| Group4, Healthy diet scores, persistent worsening | 0.457 | 1.062(0.906,1.244) |  | 0.002 | 1.278(1.095,1.490) |

Adjusted model: Adjusted for sociodemographic factors, including sex, age, marital status, nationality, education level, family economic level, region, and lifestyle factors, including smoking status, drinking status, physical activity, and dietary energy.

**Table S9.** The associations between the obesity trajectory and the change trajectory patterns of low-carbohydrate and low-fat diet scores by excluding participants with diabetes at baseline

| **Trajectories** | **Overweight trajectory vs normal weight trajectory** | |  | **Obesity trajectory vs normal weight trajectory** | |
| --- | --- | --- | --- | --- | --- |
|  | ***P* value** | ***OR* (95% *CI*)** |  | ***P* value** | ***OR* (95% *CI*)** |
| Group2, Healthy diet scores, sustained changed for the better | Reference | |  | Reference | |
| Group1, Unhealthy die score, sustained changed for the better | ＜0.001 | 1.571(1.332,1.853) |  | ＜0.001 | 1.420(1.175,1.716) |
| Group3, Moderate die score, persistent worsening | ＜0.001 | 1.448(1.223,1.715) |  | 0.014 | 1.223(1.030,1.451) |
| Group4, Healthy diet scores, persistent worsening | ＜0.001 | 1.315(1.117,1.548) |  | ＜0.001 | 1.588(1.316,1.918) |

Adjusted model: Adjusted for sociodemographic factors, including sex, age, marital status, nationality, education level, family economic level, region, and lifestyle factors, including smoking status, drinking status, physical activity, and dietary energy.

**Table S10.** The associations between the abdominal obesity trajectory and the change trajectory patterns of low-carbohydrate and low-fat diet scores by excluding participants with diabetes at baseline

| **Trajectories** | **Slowly growth of abdominal obesity trajectory vs No**  **abdominal obesity group trajectory** | |  | **Rapidly growth of abdominal obesity trajectory vs No**  **abdominal obesity group trajectory** | |
| --- | --- | --- | --- | --- | --- |
|  | ***P* value** | ***OR* (95% *CI*)** |  | ***P* value** | ***OR* (95% *CI*)** |
| Group2, Healthy diet scores, sustained changed for the better | Reference | |  | Reference | |
| Group1, Unhealthy die score, sustained changed for the better | 0.176 | 0.890(0.752,1.053) |  | ＜0.001 | 1.330(1.133,1.562) |
| Group3, Moderate die score, persistent worsening | ＜0.001 | 0.719(0.610,0.847) |  | 0.020 | 1.211(1.030,1.424) |
| Group4, Healthy diet scores, persistent worsening | 0.181 | 1.116(0.950,1.312) |  | ＜0.001 | 1.384(1.184,1.618) |

Adjusted model: Adjusted for sociodemographic factors, including sex, age, marital status, nationality, education level, family economic level, region, and lifestyle factors, including smoking status, drinking status, physical activity, and dietary energy.

**Table S11.** The associations between the obesity trajectory and the change trajectory patterns of low-carbohydrate and low-fat diet scores by excluding participants with diabetes at baseline and at all follow-up years

| **Trajectories** | **Overweight trajectory vs normal weight trajectory** | |  | **Obesity trajectory vs normal weight trajectory** | |
| --- | --- | --- | --- | --- | --- |
|  | ***P* value** | ***OR* (95% *CI*)** |  | ***P* value** | ***OR* (95% *CI*)** |
| Group2, Healthy diet scores, sustained changed for the better | Reference | |  | Reference | |
| Group1, Unhealthy die score, sustained changed for the better | ＜0.001 | 1.525(1.288,1.807) |  | 0.009 | 1.280(1.062,1.542) |
| Group3, Moderate die score, persistent worsening | ＜0.001 | 1.400(1.176,1.667) |  | 0.093 | 1.152(0.977,1.358) |
| Group4, Healthy diet scores, persistent worsening | ＜0.001 | 1.323(1.121,1.562) |  | ＜0.001 | 1.465(1.200,1.788) |

Adjusted model: Adjusted for sociodemographic factors, including sex, age, marital status, nationality, education level, family economic level, region, and lifestyle factors, including smoking status, drinking status, physical activity, and dietary energy.

**Table S12.** The associations between the abdominal obesity trajectory and the change trajectory patterns of low-carbohydrate and low-fat diet scores by excluding participants with diabetes at baseline and at all follow-up years

| **Trajectories** | **Slowly growth of abdominal obesity trajectory vs No**  **abdominal obesity group trajectory** | |  | **Rapidly growth of abdominal obesity trajectory vs No**  **abdominal obesity group trajectory** | |
| --- | --- | --- | --- | --- | --- |
|  | ***P* value** | ***OR* (95% *CI*)** |  | ***P* value** | ***OR* (95% *CI*)** |
| Group2, Healthy diet scores, sustained changed for the better | Reference | |  | Reference | |
| Group1, Unhealthy die score, sustained changed for the better | 0.127 | 0.890(0.752,1.053) |  | 0.006 | 1.330(1.133,1.562) |
| Group3, Moderate die score, persistent worsening | ＜0.001 | 0.719(0.610,0.847) |  | 0.026 | 1.211(1.03,1.424) |
| Group4, Healthy diet scores, persistent worsening | 0.079 | 1.116(0.950,1.312) |  | ＜0.001 | 1.384(1.184,1.618) |

Adjusted model: Adjusted for sociodemographic factors, including sex, age, marital status, nationality, education level, family economic level, region, and lifestyle factors, including smoking status, drinking status, physical activity, and dietary energy.
