# Supplementary material for: Association between longitudinal dietary patterns and changes in obesity: a population-based cohort study
Source: Front Public Health. 2023 Dec 1;11:1227994. doi: 10.3389/fpubh.2023.1227994 (PMC10722423; doi:10.3389/fpubh.2023.1227994)
Supplement: Supplementary file 2 [file Data_Sheet_2.docx]

**code for analyses**

**#stata trajectory**

**#Multitrajectories**

traj, multgroups(4) var1(ULCD1-ULCD6) indep1(year1-year6) model1(cnorm) max1(30) order1(1 1 1 1) var2(ULFD1-ULFD6) indep2(year1-year6) model2(cnorm) max2(30) order2(1 1 1 1) var3(HLCD1-HLCD6) indep3(year1-year6) model3(cnorm) max3(30) order3(1 1 1 1) var4(HLFD1-HLFD6) indep4(year1-year6) model4(cnorm) max4(30) order4(1 1 1 1)

multtrajplot, xtitle(year) ytitle1(ULCD) ytitle2(ULFD) ytitle3(HLCD) ytitle4(HLFD)

**#obesity trajectory**

traj, var(obesity1-obesity7) indep(year1-year7) model(cnorm) min(0) max(4) order(1 2 2)

trajplot, xtitle(year) ytitle(obesity)

**#abdominal obesity trajectory**

traj, var(abdominal_obesity1-abdominal_obesity7) indep(year1-year7) model(cnorm) min(0) max(4) order(2 2 2)

trajplot, xtitle(year) ytitle(abdominal_obesity)

**#R code**

library(haven)

data<-read_sav("total.sav")

**##Multicategorical logistic regression**

library(nnet)

library(epiDisplay)

library(haven)

library(ipw)

library(nnet)

data$new<-as.factor(data$new)

data$obesity_Group<-as.factor(data$obesity_Group)

data$abdominal_obesity_Group<-as.factor(data$abdominal_obesity_Group)

data$transfer<-as.factor(data$transfer)

data$exposure<-as.factor(data$exposure)

data$drink<-as.factor(data$drink)

data$smoke<-as.factor(data$smoke)

data$PA<-as.factor(data$PA)

data$marriage<-as.factor(data$marriage)

data$educationcat<-as.factor(data$educationcat)

data$Country<-as.factor(data$Country)

data$nationnalities<-as.factor(data$nationnalities)

data$x<-as.factor(data$x)

data$sensitive_x<-as.factor(data$sensitive_x)

data$x_sensitive<-as.factor(data$x_sensitive)

data$GENDER<-as.factor(data$GENDER)

data$x2<-as.factor(data$x2)

data$obesity_groupzhencghang<-as.factor(data$obesity_groupzhencghang)

data$abdominalobesity_Group_zhencghang<-as.factor(data$abdominalobesity_Group_zhencghang)

psModel1<-multinom(abdominalobesity_Group_zhencghang ~ exposure+age4560+GENDER+nationnalities+Country+wage+educationcat+marriage+PA

+smoke+drink+energys, data=data)

dat1<-mlogit.display(psModel1,decimal = 3)

res <- broom::tidy(psModel1)

res

regress.display (psModel1)

summary(psModel1)

res <- broom::tidy(psModel1)

res

print(res,n=30)

z <- summary(psModel1)$coefficients/summary(psModel1)$standbrd.errors

p <- (1 - pnorm(abs(z), 0, 1))*2

p

OR <- exp(coef(psModel1))

OR

OR.confi <- exp(confint(psModel1))

OR.confi

**#Multicategorical logistic regression**

library(haven)

library(ipw)

library(nnet)

data$new<-as.factor(data$new)

data$obesity_Group<-as.factor(data$obesity_Group)

data$abdominal_obesity_Group<-as.factor(data$abdominal_obesity_Group)

data$transfer<-as.factor(data$transfer)

data$exposure<-as.factor(data$exposure)

data$drink<-as.factor(data$drink)

data$smoke<-as.factor(data$smoke)

data$PA<-as.factor(data$PA)

data$marriage<-as.factor(data$marriage)

data$educationcat<-as.factor(data$educationcat)

data$Country<-as.factor(data$Country)

data$nationnalities<-as.factor(data$nationnalities)

data$x<-as.factor(data$x)

data$sensitive_x<-as.factor(data$sensitive_x)

data$x_sensitive<-as.factor(data$x_sensitive)

data$GENDER<-as.factor(data$GENDER)

data$x2<-as.factor(data$x2)

data$obesity_groupzhencghang<-as.factor(data$obesity_groupzhencghang)

data$abdominalobesity_Group_zhencghang<-as.factor(data$abdominalobesity_Group_zhencghang)

psModel2<-multinom(obesity_groupzhencghang ~ exposure+age4560+GENDER+nationnalities+Country+wage+educationcat+marriage+PA

+smoke+drink+energys, data=data)

dat2<-mlogit.display(psModel2,decimal = 3)

res <- broom::tidy(psModel2)

res

regress.display (psModel2)

summary(psModel2)

res <- broom::tidy(psModel2)

res

print(res,n=30)

z <- summary(psModel2)$coefficients/summary(psModel2)$standbrd.errors

p <- (1 - pnorm(abs(z), 0, 1))*2

p

OR <- exp(coef(psModel2))

OR

OR.confi <- exp(confint(psModel2))

OR.confi

**#Subgroup Analysis**

**#Replication of the main results in different subgroups of the population**

data1<-subset(data,data$GENDER==1)

data2<-subset(data,data$GENDER==2)

data3<-subset(data,data$age4560==1)

data4<-subset(data,data$age4560==2)

data5<-subset(data,data$age4560==3)

**#Multiple interpolation**

library(haven)

dc<-read_sav("female.sav")

dc$exposure<-as.factor(dc$exposure)

dc$drink<-as.factor(dc$drink)

dc$smoke<-as.factor(dc$smoke)

dc$PA<-as.factor(dc$PA)

dc$marriage2<-as.factor(dc$marriage2)

dc$educationcat<-as.factor(dc$educationcat)

dc$Country<-as.factor(dc$Country)

dc$nationnalities<-as.factor(dc$nationnalities)

dc$exposure<-as.factor(dc$exposure)

dc$exposure2<-as.factor(dc$exposure2)

dc$obesity_groupzhencghang<-as.factor(dc$obesity_groupzhencghang)

dc$abdominalobesity_Group_zhencghang<-as.factor(dc$abdominalobesity_Group_zhencghang)

library(mice)

library("haven")

library(lattice)

library(MASS)

library(nnet)

library(mice)

library(foreign)

str(dc)

md.pattern(dc)

imputed_Data <- mice(dc, m=5, maxit = 50, method = 'rf', seed = 500)

summary(imputed_Data)

imputed_Data$imp$nationnalities

x3<-complete(imputed_Data,action=01)

**#Inverse probability weighting**

library(tableone)

library(survey)

library(MatchIt)

library(repodataReg)

library(survival)

library(haven)

library(ipw)

w1 <-ipwpoint(

exposure = exposure,

family = "multinomial",

numerator = ~ 1,

denominator = ~ data$GENDER+ data$nationnalities + data$age4560 + data$Country + data$wage + data$educationcat + data$marriage + data$PA + data$energys + data$drink + data$smoke,

data=data)

data$w1<-w1$ipw.weights

library(nnet)

data$abdominalobesity_Group_zhencghang<-as.factor(data$abdominalobesity_Group_zhencghang)

psModel3 <- multinom(abdominalobesity_Group_zhencghang ~ exposure+age4560+GENDER+nationnalities+Country+wage+educationcat+marriage+PA

+smoke+drink+energys, data=data, model = T, weights = w1)

regress.display (psModel3)

summary(psModel3)

res <- broom::tidy(psModel3)

res

z <- summary(psModel3)$coefficients/summary(psModel3)$standbrd.errors

p <- (1 - pnorm(abs(z), 0, 1))*2

p

OR <- exp(coef(psModel3))

OR

OR.confi <- exp(confint(psModel3))

OR.confi

mlogit.display(psModel3,decimal = 3)

OR.confi <- exp(confint(psModel3))

OR.confi

OR <- exp(coef(psModel3))

OR

z <- summary(psModel3)$coefficients/summary(psModel3)$standard.errors

p <- (1 - pnorm(abs(z), 0, 1))*2

p

psModel4 <- multinom(obesity_groupzhencghang ~ exposure + nationnalities+age4560+Country+wage+educationcat+marriage+PA

+smoke+drink+energy, data=db, model = T, weights = w1)

mlogit.display(psModel4,decimal = 3)

regress.display (psModel4)

summary(psModel4)

res <- broom::tidy(psModel4)

res

OR.confi <- exp(confint(psModel4))

OR.confi

OR <- exp(coef(psModel4))

OR

z <- summary(psModel4)$coefficients/summary(psModel4)$standard.errors

p <- (1 - pnorm(abs(z), 0, 1))*2

p

**#Assignment code**

install.packages("tidyverse")

library(tidyverse)

library(haven)

df = read_sav("female.sav")

#1997 year

quantile(df$Animal_proportion_of_protein_1997,c(1/11, 2/11, 3/11, 4/11, 5/11, 6/11, 7/11, 8/11, 9/11, 10/11, 1),na.rm = T)

attach(df)

df$ULFD_AP1997[Animal_proportion_of_protein_1997 < 0.000000000 ] <- "0"

df$ULFD_AP1997[Animal_proportion_of_protein_1997 > 0.000000000 & Animal_proportion_of_protein_1997 <= 0.004391169 ] <- "1"

df$ULFD_AP1997[Animal_proportion_of_protein_1997 > 0.004391169 & Animal_proportion_of_protein_1997 <= 0.009915664 ] <- "2"

df$ULFD_AP1997[Animal_proportion_of_protein_1997 > 0.009915664 & Animal_proportion_of_protein_1997 <= 0.016054447 ] <- "3"

df$ULFD_AP1997[Animal_proportion_of_protein_1997 > 0.016054447 & Animal_proportion_of_protein_1997 <= 0.025472334 ] <- "4"

df$ULFD_AP1997[Animal_proportion_of_protein_1997 > 0.025472334 & Animal_proportion_of_protein_1997 <= 0.032847590 ] <- "5"

df$ULFD_AP1997[Animal_proportion_of_protein_1997 > 0.032847590 & Animal_proportion_of_protein_1997 <= 0.042441925 ] <- "6"

df$ULFD_AP1997[Animal_proportion_of_protein_1997 > 0.042441925 & Animal_proportion_of_protein_1997 <= 0.052696985 ] <- "7"

df$ULFD_AP1997[Animal_proportion_of_protein_1997 > 0.052696985 & Animal_proportion_of_protein_1997 <= 0.069215307 ] <- "8"

df$ULFD_AP1997[Animal_proportion_of_protein_1997 > 0.069215307 & Animal_proportion_of_protein_1997 <= 0.090120894 ] <- "9"

df$ULFD_AP1997[Animal_proportion_of_protein_1997 > 0.090120894 & Animal_proportion_of_protein_1997 <= 0.185705592 ] <- "10"

quantile(df$low_proportion_of_carbohydrate_1997,c(1/11, 2/11, 3/11, 4/11, 5/11, 6/11, 7/11, 8/11, 9/11, 10/11, 1),na.rm = T)

attach(df)

df$ULFD_LC1997[low_proportion_of_carbohydrate_1997 < 0.4343605 ] <- "0"

df$ULFD_LC1997[low_proportion_of_carbohydrate_1997 > 0.4343605 & low_proportion_of_carbohydrate_1997 <= 0.5253446 ] <- "1"

df$ULFD_LC1997[low_proportion_of_carbohydrate_1997 > 0.5253446 & low_proportion_of_carbohydrate_1997 <= 0.5808861 ] <- "2"

df$ULFD_LC1997[low_proportion_of_carbohydrate_1997 > 0.5808861 & low_proportion_of_carbohydrate_1997 <= 0.6269492 ] <- "3"

df$ULFD_LC1997[low_proportion_of_carbohydrate_1997 > 0.6269492 & low_proportion_of_carbohydrate_1997 <= 0.6596717 ] <- "4"

df$ULFD_LC1997[low_proportion_of_carbohydrate_1997 > 0.6596717 & low_proportion_of_carbohydrate_1997 <= 0.6966961 ] <- "5"

df$ULFD_LC1997[low_proportion_of_carbohydrate_1997 > 0.6966961 & low_proportion_of_carbohydrate_1997 <= 0.7293692 ] <- "6"

df$ULFD_LC1997[low_proportion_of_carbohydrate_1997 > 0.7293692 & low_proportion_of_carbohydrate_1997 <= 0.7635037 ] <- "7"

df$ULFD_LC1997[low_proportion_of_carbohydrate_1997 > 0.7635037 & low_proportion_of_carbohydrate_1997 <= 0.7958614 ] <- "8"

df$ULFD_LC1997[low_proportion_of_carbohydrate_1997 > 0.7958614 & low_proportion_of_carbohydrate_1997 <= 0.8289837 ] <- "9"

df$ULFD_LC1997[low_proportion_of_carbohydrate_1997 > 0.8289837 & low_proportion_of_carbohydrate_1997 <= 0.8809642 ] <- "10"

quantile(df$high_proportion_of_carbohydrate_1997,c(1/11, 2/11, 3/11, 4/11, 5/11, 6/11, 7/11, 8/11, 9/11, 10/11, 1),na.rm = T)

attach(df)

df$HLFD_HC1997[high_proportion_of_carbohydrate_1997 < 0.008013898 ] <- "0"

df$HLFD_HC1997[high_proportion_of_carbohydrate_1997 > 0.008013898 & high_proportion_of_carbohydrate_1997 <= 0.011778223 ] <- "1"

df$HLFD_HC1997[high_proportion_of_carbohydrate_1997 > 0.011778223 & high_proportion_of_carbohydrate_1997 <= 0.014613582 ] <- "2"

df$HLFD_HC1997[high_proportion_of_carbohydrate_1997 > 0.014613582 & high_proportion_of_carbohydrate_1997 <= 0.018656721 ] <- "3"

df$HLFD_HC1997[high_proportion_of_carbohydrate_1997 > 0.018656721 & high_proportion_of_carbohydrate_1997 <= 0.023318981 ] <- "4"

df$HLFD_HC1997[high_proportion_of_carbohydrate_1997 > 0.023318981 & high_proportion_of_carbohydrate_1997 <= 0.028785884 ] <- "5"

df$HLFD_HC1997[high_proportion_of_carbohydrate_1997 > 0.028785884 & high_proportion_of_carbohydrate_1997 <= 0.040712462 ] <- "6"

df$HLFD_HC1997[high_proportion_of_carbohydrate_1997 > 0.040712462 & high_proportion_of_carbohydrate_1997 <= 0.056282854 ] <- "7"

df$HLFD_HC1997[high_proportion_of_carbohydrate_1997 > 0.056282854 & high_proportion_of_carbohydrate_1997 <= 0.089212810 ] <- "8"

df$HLFD_HC1997[high_proportion_of_carbohydrate_1997 > 0.089212810 & high_proportion_of_carbohydrate_1997 <= 0.133478139 ] <- "9"

df$HLFD_HC1997[high_proportion_of_carbohydrate_1997 > 0.133478139 & high_proportion_of_carbohydrate_1997 <= 0.669263493 ] <- "10"

quantile(df$Plant_proportion_of_protein_1997,c(1/11, 2/11, 3/11, 4/11, 5/11, 6/11, 7/11, 8/11, 9/11, 10/11, 1),na.rm = T)

attach(df)

df$HLFD_PP1997[Plant_proportion_of_protein_1997 < 0.07959828 ] <- "0"

df$HLFD_PP1997[Plant_proportion_of_protein_1997 > 0.07959828 & Plant_proportion_of_protein_1997 <= 0.08647420 ] <- "1"

df$HLFD_PP1997[Plant_proportion_of_protein_1997 > 0.08647420 & Plant_proportion_of_protein_1997 <= 0.09168897 ] <- "2"

df$HLFD_PP1997[Plant_proportion_of_protein_1997 > 0.09168897 & Plant_proportion_of_protein_1997 <= 0.09677308 ] <- "3"

df$HLFD_PP1997[Plant_proportion_of_protein_1997 > 0.09677308 & Plant_proportion_of_protein_1997 <= 0.10131565 ] <- "4"

df$HLFD_PP1997[Plant_proportion_of_protein_1997 > 0.10131565 & Plant_proportion_of_protein_1997 <= 0.10575203 ] <- "5"

df$HLFD_PP1997[Plant_proportion_of_protein_1997 > 0.10575203 & Plant_proportion_of_protein_1997 <= 0.10892092 ] <- "6"

df$HLFD_PP1997[Plant_proportion_of_protein_1997 > 0.10892092 & Plant_proportion_of_protein_1997 <= 0.11402280 ] <- "7"

df$HLFD_PP1997[Plant_proportion_of_protein_1997 > 0.11402280 & Plant_proportion_of_protein_1997 <= 0.12087667 ] <- "8"

df$HLFD_PP1997[Plant_proportion_of_protein_1997 > 0.12087667 & Plant_proportion_of_protein_1997 <= 0.13287312 ] <- "9"

df$HLFD_PP1997[Plant_proportion_of_protein_1997 > 0.13287312 & Plant_proportion_of_protein_1997 <= 0.26358857 ] <- "10"

quantile(df$No_proportion_of_fat_1997,c(1/11, 2/11, 3/11, 4/11, 5/11, 6/11, 7/11, 8/11, 9/11, 10/11, 1),na.rm = T)

attach(df)

df$ULFD_NF1997[No_proportion_of_fat_1997 < 0.02998215 ] <- "10"

df$ULFD_NF1997[No_proportion_of_fat_1997 > 0.02998215 & No_proportion_of_fat_1997 <= 0.03715989 ] <- "9"

df$ULFD_NF1997[No_proportion_of_fat_1997 > 0.03715989 & No_proportion_of_fat_1997 <= 0.04290912 ] <- "8"

df$ULFD_NF1997[No_proportion_of_fat_1997 > 0.04290912 & No_proportion_of_fat_1997 <= 0.04942987 ] <- "7"

df$ULFD_NF1997[No_proportion_of_fat_1997 > 0.04942987 & No_proportion_of_fat_1997 <= 0.05695143 ] <- "6"

df$ULFD_NF1997[No_proportion_of_fat_1997 > 0.05695143 & No_proportion_of_fat_1997 <= 0.06522380 ] <- "5"

df$ULFD_NF1997[No_proportion_of_fat_1997 > 0.06522380 & No_proportion_of_fat_1997 <= 0.07471322 ] <- "4"

df$ULFD_NF1997[No_proportion_of_fat_1997 > 0.07471322 & No_proportion_of_fat_1997 <= 0.08518798 ] <- "3"

df$ULFD_NF1997[No_proportion_of_fat_1997 > 0.08518798 & No_proportion_of_fat_1997 <= 0.10448863 ] <- "2"

df$ULFD_NF1997[No_proportion_of_fat_1997 > 0.10448863 & No_proportion_of_fat_1997 <= 0.13183722 ] <- "1"

df$ULFD_NF1997[No_proportion_of_fat_1997 > 0.13183722 & No_proportion_of_fat_1997 <= 0.45615483 ] <- "0"

quantile(df$U_proportion_of_fat_1997,c(1/11, 2/11, 3/11, 4/11, 5/11, 6/11, 7/11, 8/11, 9/11, 10/11, 1),na.rm = T)

attach(df)

df$HLFD_UF1997[U_proportion_of_fat_1997 < 0.00000000 ] <- "10"

df$HLFD_UF1997[U_proportion_of_fat_1997 > 0.00000000 & U_proportion_of_fat_1997 <= 0.01522857 ] <- "7"

df$HLFD_UF1997[U_proportion_of_fat_1997 > 0.01522857 & U_proportion_of_fat_1997 <= 0.04110101 ] <- "6"

df$HLFD_UF1997[U_proportion_of_fat_1997 > 0.04110101 & U_proportion_of_fat_1997 <= 0.06835709 ] <- "5"

df$HLFD_UF1997[U_proportion_of_fat_1997 > 0.06835709 & U_proportion_of_fat_1997 <= 0.09068232 ] <- "4"

df$HLFD_UF1997[U_proportion_of_fat_1997 > 0.09068232 & U_proportion_of_fat_1997 <= 0.11482676 ] <- "3"

df$HLFD_UF1997[U_proportion_of_fat_1997 > 0.11482676 & U_proportion_of_fat_1997 <= 0.14879971 ] <- "2"

df$HLFD_UF1997[U_proportion_of_fat_1997 > 0.14879971 & U_proportion_of_fat_1997 <= 0.19946579 ] <- "1"

df$HLFD_UF1997[U_proportion_of_fat_1997 > 0.19946579 & U_proportion_of_fat_1997 <= 0.47720291 ] <- "0"
